# The function of the ZFP189 transcription factor in the nucleus accumbens facilitates cocaine-specific transcriptional and behavioral adaptations

**DOI:** 10.1038/s41380-024-02852-7

**Published:** 2024-11-25

**Authors:** Joseph A. Picone, Annalise Hassan, R. Kijoon Kim, Diego Piñeiro Lira, Gabriella M. Silva, Natalie L. Truby, Hadessah Y. Johnson, Collin D. Teague, Rachael L. Neve, Matthew L. Banks, Xiaohong Cui, Peter J. Hamilton

**Affiliations:** 1 Department of Anatomy and Neurobiology, Virginia Commonwealth University School of Medicine, Richmond, VA USA; 2 Department of Pharmacology and Toxicology, Virginia Commonwealth University School of Medicine, Richmond, VA USA; 3 Nash Family Department of Neuroscience and Friedman Brain Institute, Icahn School of Medicine at Mount Sinai, New York, NY USA; 4 Gene Delivery Technology Core, Massachusetts General Hospital, Cambridge, MA USA

**Keywords:** Neuroscience, Molecular biology

## Abstract

Distinguishing the brain mechanisms affected by distinct addictive drugs may inform targeted therapies against specific substance use disorders (SUDs). Here, we explore the function of a drug-associated, transcriptionally repressive transcription factor (TF), ZFP189, whose expression in the nucleus accumbens (NAc) facilitates cocaine-induced molecular and behavioral adaptations. To uncover the necessity of ZFP189-mediated transcriptional control in driving cocaine-induced behaviors, we created synthetic ZFP189 TFs of distinct transcriptional function, including ZFP189^VPR^, which activates the expression of target genes and exerts opposite transcriptional control to the endogenously repressive ZFP189. By virally delivering synthetic ZFP189 TFs to the NAc of mice, we discover that the transcriptional control exerted by synthetic or endogenous ZFP189 solely alters behavioral adaptations to cocaine but not morphine, saline, or sucrose. Further, these synthetic ZFP189 TFs are only capable of producing gene-expression changes in rodents exposed to cocaine, but not morphine or saline. In these cocaine exposed mice, the gene-expression profile produced by ZFP189^VPR^ is inversely related to the cocaine-induced transcriptional response, as characterized by Upstream Regulator Analysis in Ingenuity Pathway Analysis. Lastly, we demonstrate that NAc ZFP189^WT^ increases vulnerability to cocaine reinforcement through selective sensitization to the reinforcing effects of small cocaine doses. In contrast, ZFP189^VPR^ treated mice do not experience changes in cocaine sensitivity and had lower rates of cocaine self-administration. Collectively, this research describes the brain mechanisms by which a TF specifically coordinates the molecular adaptations that produce increased cocaine addiction-like behaviors. The use of synthetic ZFP189^VPR^ uncovers novel strategies for therapeutic interventions to potentially halt these cocaine-induced transcriptional processes.

## Introduction

Substance use disorders (SUDs) are driven by drug-induced brain molecular adaptations that contribute to the escalation of drug-taking behaviors over time [1–3]. SUD research has identified brain physiological mechanisms that are common among many misused drugs, such as increases in extracellular dopamine in the nucleus accumbens (NAc), an important brain reward region [4–7]. However, drugs with addictive potential vary widely in chemical structure, pharmacokinetics, and impact on behavior. Thus, distinct brain molecular adaptations likely exist for distinct drugs of abuse, and an increased resolution of the brain mechanistic responses to distinct drug classes may illuminate novel avenues for targeted therapies for specific SUDs. There is a need for therapeutic options for SUD since, unlike opioid use disorder (OUD) for which there are currently three Food and Drug Administration (FDA)-approved pharmacotherapies (e.g., naltrexone, buprenorphine, and methadone) [8], there are currently zero FDA-approved pharmacotherapies available for individuals suffering from cocaine use disorder (CUD).

Our team has identified NAc expression of ZFP189 as contributing to behavioral and physiological adaptations to cocaine use [9]. In this prior work, we applied CRISPR-mediated gene activation and gene repression directed to the *Zfp189* gene within the mouse NAc and measured effects on cocaine- and morphine-related behaviors. We discovered that *Zfp189* expression in the NAc uniquely affected cocaine-related behaviors, but not morphine-related behaviors [9]. These discoveries led us to hypothesize that the drug-induced activation of *Zfp189* within the NAc uniquely participates in the brain adaptations that facilitate the progression of CUD-like behaviors. This prior research focused on the molecular events that control NAc *Zfp189* gene expression but did not illuminate the “downstream” molecular functions of the ZFP189 TF gene product. A clearer understanding of the molecular action of the ZFP189 TF may illuminate novel druggable targets to specifically treat CUD.

Here, we aimed to characterize the drug-associated functions of the *Zfp189* gene product, ZFP189, which is a Krüppel-associated box (KRAB) zinc finger protein (KZFP) TF. We replaced the endogenous repressive KRAB moiety of wild-type ZFP189 (ZFP189^WT^) with the transcriptional activator VP64-p65-Rta (VPR) to create the novel synthetic TF ZFP189^VPR^. We also removed the KRAB moiety entirely, generating a functionally inert, control TF with no functional domain (NFD; ZFP189^NFD^). We virally delivered these synthetic ZFP189 TFs in mouse NAc. We discovered that ZFP189 TFs augmented drug-related behaviors for cocaine, but did not affect morphine-, saline-, or food-related behaviors. We discovered that ZFP189 TFs can only exert transcriptional control in the NAc of mice that have been exposed to cocaine, and the consequence of this transcriptional control was to regulate genes involved in synaptic plasticity. Further, using mouse cocaine intravenous self-administration (IVSA) we discovered that NAc ZFP189^WT^ facilitates drug taking particularly at small cocaine doses. Collectively, this research demonstrates that NAc ZFP189 causally drives the molecular and behavioral responses to cocaine.

## Results

### Synthetic ZFP189 TFs exert opposing gene-regulatory function at a target gene in vitro

**Fig. 1:**
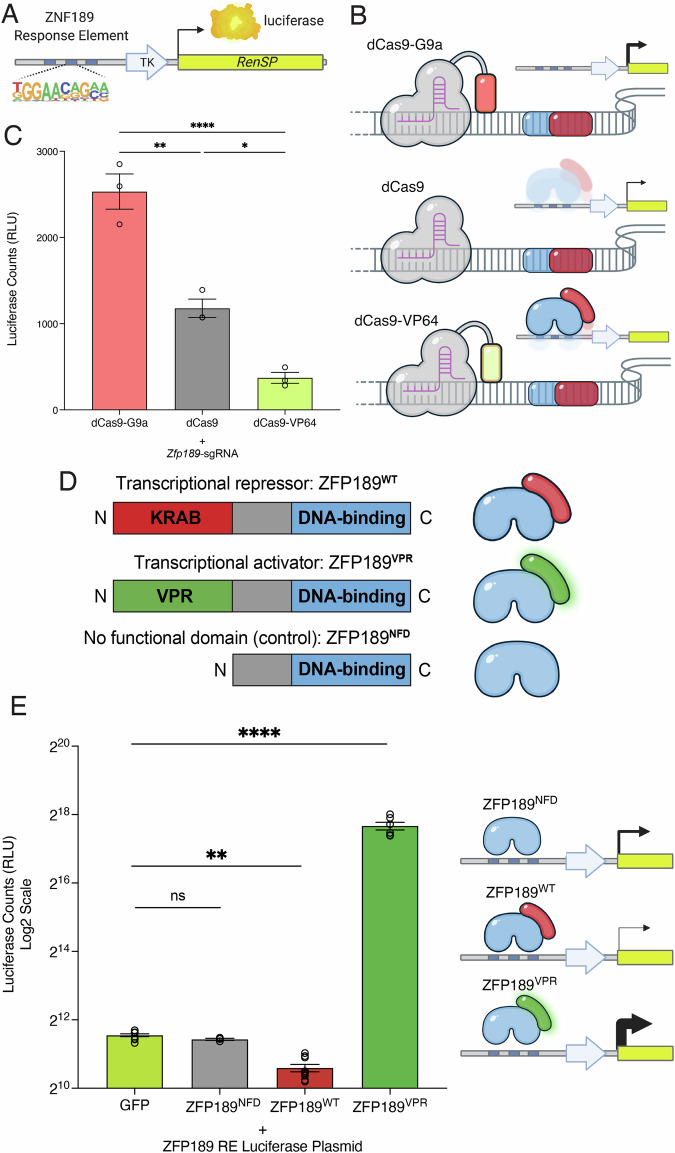
ZFP189 is naturally a repressive transcription factor and ZFP189 transcriptional control can be inverted to gene-activation with synthetic ZFP189^VPR^. **A** Graphic representation of the ZFP189 DNA response element (RE) luciferase plasmid reporter target gene. **B** Graphic representation of CRISPR-dCas9 constructs delivered to the ZFP189 DNA response element via a *Zfp189* guide RNA; dCas9-G9a (transcriptional repressor), dCas9 (control), and dCas9-VP64 (transcriptional activator). **C** Transfection of N2a cells with CRISPR-dCas9 constructs reveals that repression of *Zfp189* induced significant increases in luciferase expression, whereas activation of *Zfp189* induced significant decreases in luciferase expression. This suggests that the endogenous ZFP189 TF is functioning as a transcriptional repressor. Ordinary one-way ANOVA (Tukey Test); *p-value < 0.05, **p-value < 0.005, ****p-value < 0.0001, *n* = 3 wells per condition. **D** Graphic representation of the three ZFP189 TF constructs including: ZFP189^WT^ which is identical to the endogenously expressed protein which contains a transcriptionally repressive N-terminal Krüppel associated box (KRAB) domain and a C-terminal Cys_2_-His_2_ DNA-binding domain; ZFP189^VPR^ in which the endogenous KRAB domain is replaced with the transcriptional activator VP64-p65-Rta (VPR); and ZFP189^NFD^ in which any transcriptional regulatory domain is entirely removed, which serves as a control. **E** These tools were validated by co-transfecting a rodent neuroblastoma Neuro2a cell line with both the ZFP189 RE luciferase plasmid and iterations of our ZFP189 TFs, with *luciferase* expression showing increases produced by ZFP189^VPR^ and significant decreases produced by ZFP189^WT^ relative to the GFP control groups. Ordinary one-way ANOVA (Tukey Test); ns *p*-value > 0.05, **p-value < 0.01, ****p-value < 0.0001, *n* = 6–9 wells per condition.

To assay potential ZFP189 TF function, we created a *luciferase* reporter construct. We inserted the experimentally determined DNA response element (RE) of the human ortholog ZNF189 (92% identical to ZFP189 on the amino acid level) [10] upstream of the thymidine kinase (TK) promoter which drives *luciferase* reporter gene expression (Fig. 1A). Alongside a validated and previously published *Zfp189*-targeting sgRNA [9, 11], we directed CRISPR-mediated *Zfp189* activation, with dCas9-VP64, or CRISPR-mediated repression, with dCas9-G9a, in mouse neuroblastoma Neuro-2a (N2a) cells (Fig. 1B). We observe that CRISPR-mediated *Zfp189* gene activation increases *Zfp189* mRNA (quantified by qRT-PCR and normalized to dCas9 control, *Zfp189* expression relative to the geometric mean of *Gapdh, Actin*, and *Hprt1* expression (data not plotted); dCas9 + *Zfp189*-sgRNA: 1.00 ± 0.026; dCas9-VP64 + *Zfp189*-sgRNA: 4.52 ± 0.33; *n* = 3–6; *p* < 0.05 by Student’s *t*-test) and decreases ZFP189-sensitive *luciferase* expression (Fig. 1C). Oppositely, dCas9-G9a mediated *Zfp189* gene repression increases *luciferase* expression (Fig. 1C). This indicates that the *Zfp189* gene product is a TF of gene-repressive function, which is consistent with the known transcriptionally repressive function of other members of the KZFP TF family [12–14].

We next sought to uncover the mechanisms by which drug exposure might regulate *Zfp189* expression and function. In N2a cells bath application of either cocaine or morphine-induced dose dependent increases in *Zfp189* mRNA expression (Supplementary Fig. 1A, B), ZFP189 protein accumulation (Supplementary Fig. 1C, D), and gene-repressive function at the ZFP189-sensitive *luciferase* reporter gene (Supplementary Fig. 1E, F). In pre-incubating these N2a cells with dopamine receptor antagonists (SCH-23390 and sulpiride) or opioid receptor antagonists (naloxone), and again applying either cocaine or morphine as before, we again see drug dose dependent increases in *Zfp189* mRNA, ZFP189 protein, and ZFP189 function (Supplementary Fig. 2). This indicates that both cocaine and morphine can activate *Zfp189* expression and function in a neuroblastoma cell line, and this activation can occur independently from signaling through dopamine or opioid receptors.

Furthermore, we have previously determined that cocaine exposure increases *Zfp189* expression in rodent NAc at acute but not chronic timepoints [9]. Here, we subjected mice to an identical dosing schedule of morphine injections, and discovered that morphine similarly activates *Zfp189* gene expression in the NAc with significant increases at the acute but not chronic timepoint (Supplementary Fig. 3). Collectively, these data indicate that both cocaine and morphine exposure activate *Zfp189* gene expression in cell culture and within intact brain reward regions, illustrating the highly drug-responsive nature of the *Zfp189* gene.

To more thoroughly investigate the functions of the *Zfp189* gene product, we created synthetic ZFP189 TFs of distinct transcriptional function (Fig. 1D). We synthesized three novel ZFP189 TFs. ZFP189^WT^, which is identical to the endogenous ZFP189 protein and contains the N-terminal repressive KRAB domain and a C-terminal Cys_2_-His_2_ DNA-binding domain (Fig. 1D, top); ZFP189^VPR^, wherein the endogenous KRAB domain is replaced with the synthetic transcriptional activator VPR (Fig. 1D, middle); and ZFP189^NFD^ in which any transcriptional regulatory domain is entirely removed which serves to control for any non-specific effects of the expression vector or the over-expressed ZFP189 DNA-binding domain (Fig. 1D, bottom). These synthetic ZFP189 TFs have been introduced and thoroughly validated in our other work in the prefrontal cortex (PFC) [15]. By co-transfecting mouse N2a cells with both the ZFP189 RE *luciferase* plasmid and ZFP189 TFs, we observed that ZFP189^VPR^ induces robust targeted gene activation, ZFP189^WT^ induces gene-targeted repression, and ZFP189^NFD^ exerts no regulatory control (Fig. 1E). Further when we removed the ZFP189 RE motifs from our *luciferase* reporter plasmid, we saw no change in ZFP189^NFD^ gene-regulatory function (Supplementary Fig. 4A), we see that the repressive function of ZFP189^WT^ is released (Supplementary Fig. 4B), and the gene-activating function of ZFP189^VPR^ is dramatically reduced (Supplementary Fig. 4C). These data indicate that the transcriptional regulatory effects of our synthetic ZFP189 TFs are dependent on accessibility to gene-proximal DNA binding sites. Also, the null effect of ZFP189^NFD^ in Fig. 1E and Supplemental Fig. 4A supports its use as an appropriate control to account for any off-target biological effects of our synthetic ZFP189 TFs distinct from the transcriptional control exerted by the ZFP189 functional moiety. Lastly, we synthesized the VPR moiety alone to determine the extent to which ZFP189^VPR^ luciferase gene activation is due to non-specific VPR activity (Supplementary Fig. 4D). We observe that free VPR does not activate *luciferase* expression beyond the level of the GFP control, whereas VPR fused to the ZFP189 DNA-binding domain (ZFP189^VPR^) activates *luciferase* gene expression (Supplementary Fig. 4E). Collectively, these data indicate that we are capable of utilizing these synthetic ZFP189 TFs to bi-directionally regulate the expression of ZFP189 target genes, and that the gene regulatory function of these synthetic ZFP189 TFs requires physical access to the DNA, facilitated both by the ZFP189 DNA-binding domain and accessibility to gene-proximal ZFP189 RE motifs.

### NAc expressed synthetic ZFP189 TFs regulate behavioral and transcriptional responses to cocaine, but not morphine

**Fig. 2:**
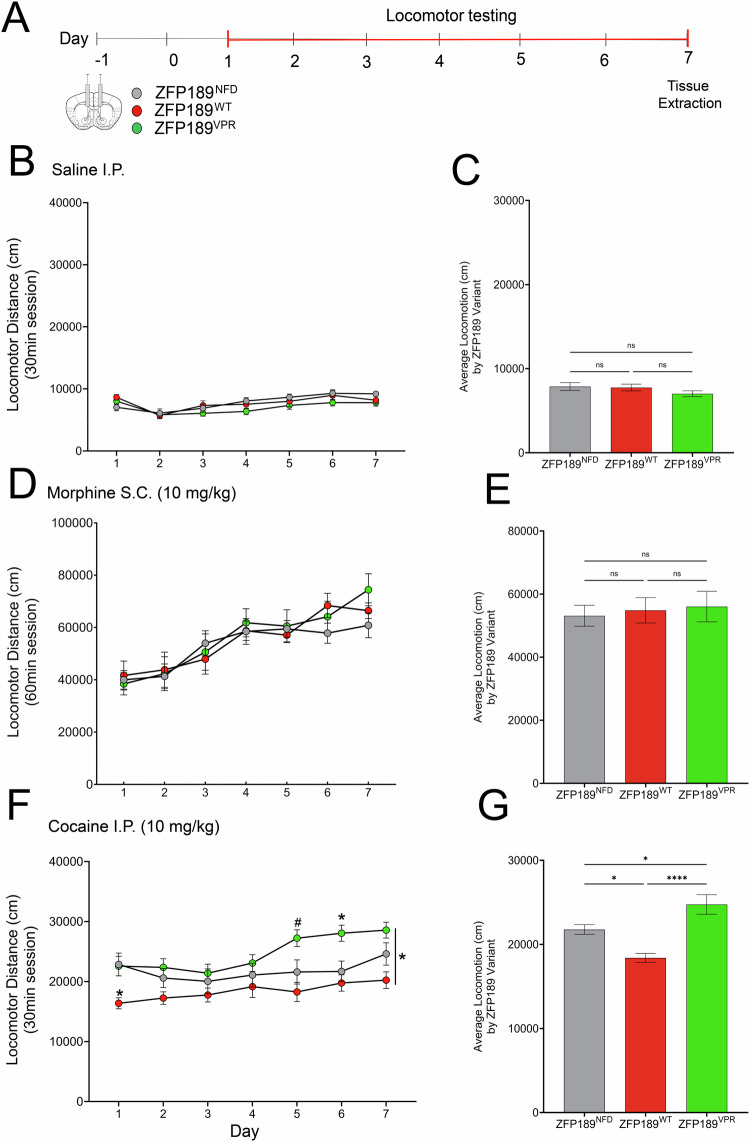
Viral delivery of synthetic ZFP189 transcription factors to mouse NAc regulates locomotor responses to cocaine, but not morphine or saline. **A** Graphic representation of experimental timeline. Mice had ZFP189 TFs virally delivered to NAc and given a day to rest before seven-day locomotor sensitization trials. **B** Daily locomotor responses, by ZFP189 TF viral treatment, measured as distance moved (cm) following a saline injection. No significant effects observed. Repeated measures Two-way ANOVA (Tukey Test); *p*-value > 0.05. *n* = 11 (ZFP189^NFD^), *n* = 12 (ZFP189^WT^) and *n* = 12 (ZFP189^VPR^) mice. **C** Average distance moved (cm), by ZFP189 TF viral treatment, across the seven-day saline locomotor assay. No significant effects observed. Ordinary one-way ANOVA (Tukey Test); ns *p*-value > 0.05. *n* = 11 (ZFP189^NFD^), *n* = 12 (ZFP189^WT^) and *n* = 12 (ZFP189^VPR^) mice. **D** Daily locomotor responses, by ZFP189 TF viral treatment, measured as distance moved (cm) following a 10 mg/kg morphine injection. No significant effects observed. Repeated measures Two-way ANOVA (Tukey Test); *p*-value > 0.05. *n* = 11 (ZFP189^NFD^), *n* = 12 (ZFP189^WT^) and *n* = 12 (ZFP189^VPR^). **E** Average distance moved (cm), by ZFP189 TF viral treatment, across the seven-day morphine-induced locomotor assay. No significant effects observed. Ordinary one-way ANOVA (Tukey Test); ns *p*-value > 0.05. *n* = 11 (ZFP189^NFD^), *n* = 12 (ZFP189^WT^) and n = 12 (ZFP189^VPR^). **F** Daily locomotor responses, by ZFP189 TF viral treatment, measured as distance moved (cm) following a 10 mg/kg cocaine injection. There was a significant interaction between viral treatment and time, denoted by the vertical bar and asterisk. ZFP189^VPR^ induced significant increases, and ZFP189^WT^ induced significant decreases, in locomotion on individual days relative to ZFP189^NFD^. Repeated measures Two-way ANOVA (Tukey Test); #*p*-value < 0.1, *p-value < 0.05. *n* = 11 (ZFP189^NFD^), *n* = 12 (ZFP189^WT^) and *n* = 12 (ZFP189^VPR^). **G** Average distance moved (cm) in response to cocaine by ZFP189 TF groups averaged across seven days. ZFP189^VPR^ significantly increased locomotion, while ZFP189^WT^ significantly decreased locomotion, relative to ZFP189^NFD^. Ordinary one-way ANOVA (Tukey Test); *p-value < 0.05, ***p-value < 0.005. *n* = 11 (ZFP189^NFD^), *n* = 12 (ZFP189^WT^) and *n* = 12 (ZFP189^VPR^).

We next sought to uncover the consequences of NAc ZFP189-mediated transcriptional control on drug-related behaviors. To be consistent with our earlier studies [9], and to leverage the rapid *trans-*gene expression [16] which is likely necessary to uncover the transcriptional consequences of our synthetic ZFP189 TFs, we packaged the constructs in herpes simplex viruses (HSVs). We virally delivered each of the synthetic ZFP189 TFs bi-laterally to the NAc and recorded the acute locomotor response to daily injections of saline, morphine (10 mg/kg), or cocaine (10 mg/kg) (Fig. 2A). NAc-delivered ZFP189 TFs possessing our three varied transcriptional moieties had no effect on saline- (Fig. 2B, C) or morphine- (Fig. 2D, E) elicited locomotor responses. In contrast, ZFP189 TFs had divergent effects on locomotor responses to cocaine (Fig. 2F, G). When comparing the total daily distance moved, ZFP189^WT^ and ZFP189^VPR^ repress and potentiate behavioral locomotor responses to cocaine (Fig. 2G) in a pattern similar to their opposite transcriptional regulatory control in vitro (Fig. 1E). In sum, these data indicate that some feature of cocaine exposure, but not saline or morphine exposure, renders the NAc ZFP189 TFs capable of augmenting behavioral responses to drug experience. Further, synthetic ZFP189 TFs of opposite transcriptional function drive similarly opposing behavioral responses to cocaine experience.

**Fig. 3:**
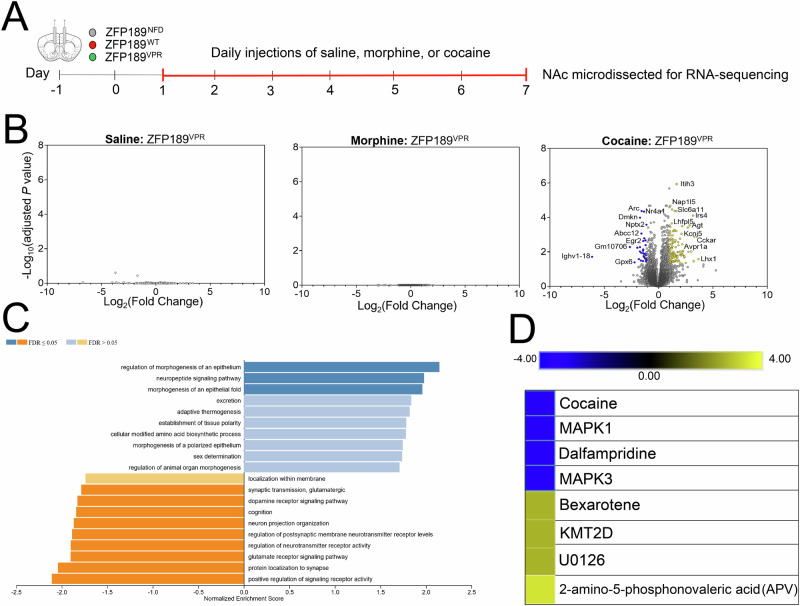
NAc ZFP189^VPR^ only regulates gene-expression in mice with a history of cocaine exposure and impedes the canonical cocaine-induced transcriptional response. **A** Timeline of experimental design preceding RNA-sequencing (RNAseq). A representative subset of mice from Fig. 2 were selected and RNAseq was performed on virally-manipulated NAc tissues. Behavioral data from these mice are in Supp. Fig. 5. For each RNAseq analysis, differentially expressed genes (DEGs) were generated relative to ZFP189^NFD^ mice from the same treatment group. **B** RNAseq volcano plots depicting the transcriptional consequence of NAc ZFP189^VPR^ expression in mice with a history of saline (left panel), morphine (middle panel), or cocaine (right panel) injections. ZFP189^VPR^ does not of regulate gene expression in the NAc of mice treated with saline or morphine. ZFP189^VPR^ regulates both up- and downregulated DEGs in mice that have been treated with cocaine (DEGs generated relative to ZFP189^NFD^ within all treatment conditions, DEG significance cutoff is 5% FDR adjusted *p*-value < 0.05 and Log2 Fold Change>1.0, *n* = 5 mice per condition in each comparison). **C** Gene set enrichment analysis (GSEA) analysis on entire cocaine-induced DEG lists from panel (**B**). Top upregulated terms were involved in neuropeptide signaling pathways, whereas top downregulated terms were involved in synaptic signaling. **D** Ingenuity Upstream Regulator analysis performed in Ingenuity Pathway Analysis (IPA) specifically on the significant cocaine-induced DEGs from Panel (**B**) reveal the transcriptional regulators that could most explain the ZFP189^VPR^ regulated DEGs. Cocaine is the top of four upstream regulators most anti-correlated with ZFP189^VPR^ regulated gene-expression profiles, whereas 2-amino-5-phosphonovaleric acid (APV), an NMDA antagonist, is the top of four upstream regulators whose function could most explain our DEG lists.

To uncover the transcriptional basis for these behavioral results, immediately following locomotor testing on day seven, mice were sacrificed, virally manipulated NAc tissues were micro-dissected, and individual mouse NAc tissues were subjected to RNAseq (Fig. 3; behaviors of mice used for RNAseq in Supplementary Fig. 5 and complete lists of differentially expressed genes (DEGs) for all comparisons in Supplementary Table 1). We generated DEGs relative to the drug-exposure-matched ZFP189^NFD^ condition following repeated treatments with saline, morphine, or cocaine (Fig. 3A). We observed that ZFP189^VPR^ produced no significant DEGs in the NAc of mice exposed to saline (Fig. 3B, left) or morphine (Fig. 3B, middle). However, in mice exposed to cocaine, ZFP189^VPR^ we detected many significantly up- and downregulated DEGs (Fig. 3B, right). We similarly performed RNAseq on the NAc of mice manipulated with ZFP189^WT^ and performed rank-rank hypergeometric overlap (RRHO) analyses to compare the transcriptional consequences of these ZFP189 TFs in mice exposed to saline, morphine, or cocaine. We again see that only cocaine exposure produces strong patterns of gene-regulatory control in NAc tissues manipulated with these ZFP189 TFs (Supplementary Fig. 6), further supporting the notion that the function of ZFP189 depends on the drug experience of the animal.

To characterize the functions of genes that are impacted by ZFP189^VPR^ in the context of cocaine, we performed gene set enrichment analysis (GSEA) on the entire cocaine-associated DEG lists from Fig. 3B. ZFP189^VPR^ regulated DEGs showed enrichment of terms associated with neuropeptide signaling and negative enrichment of terms associated with small-molecule neurotransmission (Fig. 3C). Next, we employed Upstream Regulator Analysis (URA) in Ingenuity Pathway Analysis (IPA) to identify and contextualize potential transcriptional regulators whose functions could explain our observed significant DEGs. The IPA URA predicts relationships between hypothetical molecular upstream regulators and experimentally-determined transcriptional response and is derived from the pre-existing literature compiled in the Ingenuity Knowledge Base [17]. The predicted upstream regulator that could most explain the ZFP189^VPR^-driven transcriptional response in cocaine-exposed mice was 2-amino-5-phosphonovaleric acid (APV), an NMDA antagonist, whereas the top predicted upstream regulator that was most anti-correlated with this ZFP189^VPR^-driven gene-expression profile was cocaine itself (Fig. 3D). Thus, despite the fact that these mice were repeatedly injected with cocaine, the transcriptional consequence of NAc ZFP189^VPR^ function is to generate a transcriptional profile largely opposite to the known transcriptional response to cocaine, as deduced by IPA URA.

### NAc ZFP189 specifically augments cocaine reward and self-administration behaviors

**Fig. 4:**
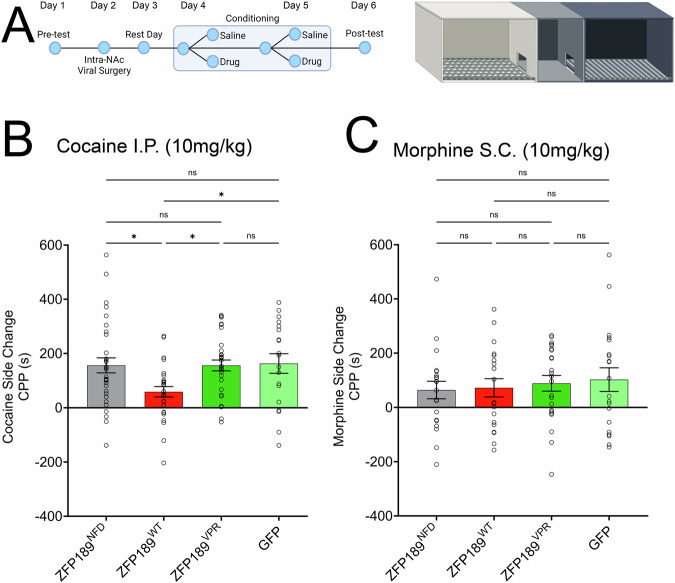
NAc ZFP189 function alters conditioned place preference to cocaine without affecting conditioned place preference to morphine, at a 10 mg/kg dose. **A** Experimental timeline for cocaine and morphine conditioned place preference (CPP). Days are numbered above the timeline. **B** CPP scores are reported as change in time spent in the drug-paired side of the conditioning chamber after versus before drug conditioning (i.e., time in drug paired chamber post-conditioning minus time in drug paired chamber pre-conditioning). Mice manipulated intra-NAc with ZFP189^WT^ and conditioned with 10 mg/kg cocaine I.P. show lowest CPP response relative to the other viral treatment groups. Ordinary one-way ANOVA (Tukey Test); ns *p*-value > 0.05, *p-value < 0.05. *n* = 32 (ZFP189^NFD^), *n* = 28 (ZFP189^WT^), *n* = 31 (ZFP189^VPR^), and *n* = 19 (GFP) mice. **C** No significant differences were observed as a result of viral treatment in a 10 mg/kg morphine CPP. Ordinary one-way ANOVA (Tukey Test); ns *p*-value > 0.05. *n* = 21 (ZFP189^NFD^), *n* = 20 (ZFP189^WT^), *n* = 23 (ZFP189^VPR^), and *n* = 20 (GFP) mice.

We next characterized NAc ZFP189 function in drug related behaviors. We virally manipulated mice intra-NAc with ZFP189 TFs and performed conditioned place preference (CPP) for either cocaine or morphine (Fig. 4A). We discovered that mice manipulated intra-NAc with ZFP189^WT^ have reduced cocaine CPP behavioral responses relative to GFP, ZFP189^NFD^, and ZFP189^VPR^ manipulated mice (Fig. 4B). The reduction in cocaine CPP in ZFP189^WT^ treated mice is consistent with our earlier published work in which CRISPR-mediated activation of *Zfp189* decreases cocaine CPP [9]. Importantly, no ZFP189 TF treatment affected morphine CPP (Fig. 4C), further indicating that NAc expression of these ZFP189 TFs do not modulate morphine reward related behaviors.

**Fig. 5:**
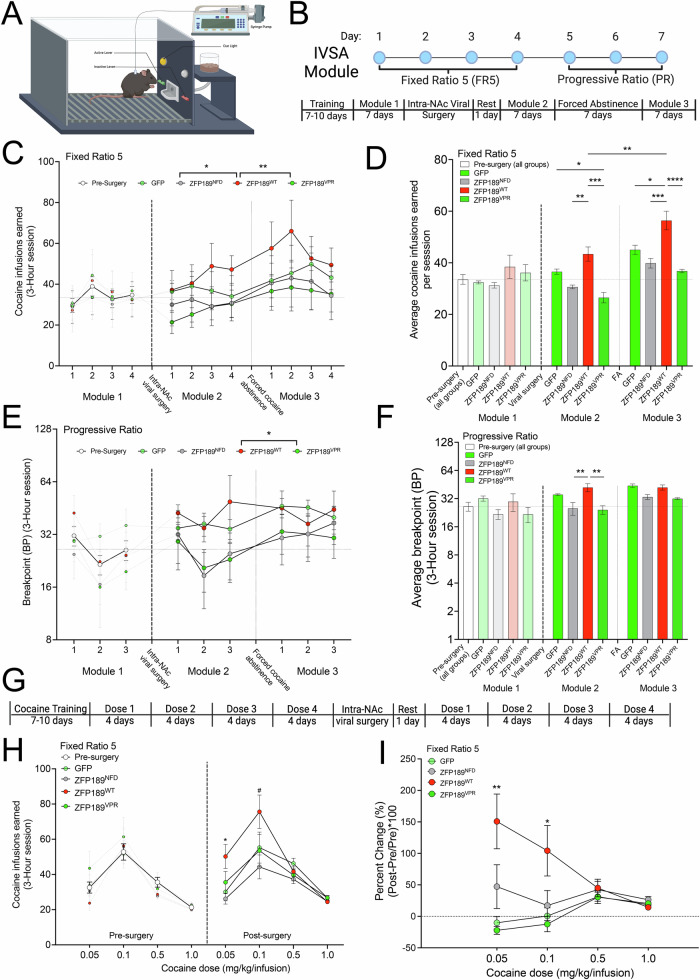
NAc ZFP189^WT^ increases rates of cocaine self-administration and drives sensitization to cocaine reinforcement. **A** Cartoon depiction of the intravenous self-administration (IVSA) operant chamber. **B** Timeline of the experimental design. Briefly, mice were trained to respond under a fixed-ratio (FR) 1 schedule for 0.5 mg/kg/infusion cocaine until stable responding was achieved within 7–10 daily three-hour sessions. We then employed seven-day modules to sequentially test FR5 and progressive ratio (PR) responding for 0.5 mg/kg/infusion cocaine in daily three-hour sessions. Module 1 preceded intra-NAc surgery, module 2 immediately followed viral surgery, and module 3 occurred after a seven-day period of forced cocaine abstinence. **C** The number of cocaine infusions are plotted during each FR5 session of the behavioral module. Horizontal dotted line denotes collective pre-surgery baseline behavior. Over the course of all post-surgery sessions, there was a main effect of viral treatment, with ZFP189^WT^ treated mice self-administering significantly more cocaine infusions than ZFP189^NFD^ and ZFP189^VPR^. Repeated measures two-way ANOVA (Tukey Test); *p-value < 0.05, **p-value < 0.01. *n* = 5 (GFP), *n* = 5 (ZFP189^NFD^), *n* = 5 (ZFP189^WT^) and *n* = 5 (ZFP189^VPR^) mice. **D** The number of cocaine infusions earned during an FR5 session, averaged by viral treatment group over the course of an individual module. Horizontal dotted line denotes collective pre-surgery baseline behavior. In module 2, ZFP189^VPR^ treated mice self-administered fewer cocaine infusions relative to GFP or ZFP189^WT^ mice. Following seven days of forced cocaine abstinence (FA), ZFP189^WT^ treated mice showed the highest level of cocaine self-administration relative to all other viral groups. Further, ZFP189^WT^-treated mice showed a significant increase in cocaine self-administration following FA when comparing infusions in module 2 to module 3. Ordinary one-way ANOVA (Tukey Test); ns *p*-value > 0.05, *p-value < 0.05, **p-value < 0.01, ***p-value < 0.001 ****p-value < 0.0001. *n* = 5 (GFP), *n* = 5 (ZFP189^NFD^), *n* = 5 (ZFP189^WT^) and *n* = 5 (ZFP189^VPR^) mice. **E** The PR break points (BPs), which correspond to the highest achieved number of active lever presses that led to a cocaine infusion (see Methods), are reported per module PR session. Horizontal dotted line denotes collective pre-surgery baseline behavior. Over the course of all post-surgery sessions, there was a main effect of viral treatment, but the only viral treatment comparison that significantly differed was the ZFP189^WT^ to ZFP189^VPR^ comparison. Importantly, in accordance with statistical analyses for PR BPs that account for the homogeneity of the variance [58], statistical analyses are performed on the infusions earned during each PR session, while PR BPs are plotted in the figure. Repeated measured two-way ANOVA (Tukey Test); *p-value < 0.05. *n* = 5 (GFP), *n* = 5 (ZFP189^NFD^), *n* = 5 (ZFP189^WT^) and *n* = 5 (ZFP189^VPR^) mice. **F** Cocaine BPs averaged within an individual module for each viral treatment group. Horizontal dotted line denotes collective pre-surgery baseline behavior. In analyzing infusions earned in the PR module [58], ZFP189^WT^-treated mice in module 2 were different than ZFP189^NFD^- and ZFP189^VPR^-treated mice. Ordinary one-way ANOVA (Tukey Test); ns *p*-value > 0.05. *n* = 5 (GFP), *n* = 5 (ZFP189^NFD^), *n* = 5 (ZFP189^WT^) and *n* = 5 (ZFP189^VPR^) mice. **G** Timeline of dose effect experiment. Briefly, mice were trained to respond for 0.5 mg/kg/infusion cocaine under an FR1 schedule until stable responses were achieved within 7–10 daily three-hour sessions. Subsequently, one of four cocaine doses (0.05, 0.1, 0.5, and 1.0 mg/kg/infusion) were tested in sequential 3-h sessions under an FR5 schedule both before and after viral manipulation. **H** Dose effect functions on the left represent data prior to intra-NAc viral surgery, whereas dose effect functions on the right were generated post-surgery. There was a significant dose x viral treatment group interaction. Repeated measures two-way ANOVA (Tukey Test); # *p*-value < 0.1, *p-value < 0.05. *n* = 5 (GFP), *n* = 5 (ZFP189^NFD^), *n* = 5 (ZFP189^WT^) and *n* = 5 (ZFP189^VPR^) mice. ZFP189^WT^ earned significantly more cocaine infusions at the 0.05 mg/kg/infusion dose and trended towards significantly higher cocaine consumption at the 0.1 mg/kg/infusion dose. **I** Percent change in cocaine infusions earned post-surgery, calculated as (Post-Pre/Pre)*100. The ZFP189^WT^ group showed significantly higher positive percent change at the 0.05 and 0.1 mg/kg/infusion cocaine doses relative to both the GFP and the ZFP189^VPR^ groups. Ordinary two-way repeated measures ANOVA (Tukey test); *p-value < 0.05, **p-value < 0.01. *n* = 5 (GFP), *n* = 5 (ZFP189^NFD^), *n* = 5 (ZFP189^WT^) and *n* = 5 (ZFP189^VPR^) mice. Vertical dashed lines denote surgery. Horizontal dotted lines denote the pre-surgery average as a baseline. In panels **C**, **E**, and **H**, all pre-surgery values were combined in the white dots, alongside faint representations of the group averages prior to surgery.

Next, we explored what consequence these ZFP189 TFs have on volitional cocaine-taking behaviors via cocaine IVSA. Mice were trained to self-administer 0.5 mg/kg/infusion cocaine under a fixed-ratio 1 (FR1) schedule of reinforcement during daily 3-h sessions. Upon stable responding on the cocaine-paired active lever, mice entered a behavioral IVSA module that consisted of four sequential FR5 self-administration sessions followed by three sequential progressive ratio (PR) self-administration sessions with all sessions lasting 3 h and 0.5 mg/kg/infusion cocaine availability (Fig. 5A, B). We characterized baseline IVSA infusions, delivered our ZFP189 TFs intra-NAc, performed the behavioral IVSA module described above immediately following viral delivery, and then performed a third IVSA module following a seven-day cocaine forced abstinence (FA) (Fig. 5B). We discovered that ZFP189^WT^-treated mice emitted more lever responses to produce cocaine infusions across the behavioral modules following viral delivery and increased their cocaine self-administration following a period of FA (Fig. 5C, D). Alternatively, ZFP189^VPR^-treated mice self-administered fewer cocaine infusions in the behavioral module immediately following viral delivery (Fig. 5D). In the collective PR modules following viral surgery, the only viral treatment effects that differed was the ZFP189^WT^ to ZFP189^VPR^ comparison (Fig. 5E), and ZFP189^WT^-treated mice produced more cocaine infusions on a PR schedule than ZFP189^NFD^- or ZFP189^VPR^-treated mice in the behavioral module immediately following viral surgery (Fig. 5F). These data suggest that NAc ZFP189^WT^ function increases rates of cocaine self-administration.

To more completely characterize this ZFP189^WT^-driven increase in cocaine self-administration, we performed a cocaine IVSA dose-effect function in an independent cohort both before and after viral manipulation (Fig. 5G). Before viral manipulation, cocaine displayed the prototypic inverted U-shaped dose-effect function (Fig. 5H, left). Intra-NAc ZFP189^WT^ treatment resulted in a selective vertical shift in the ascending limb of the dose-effect function (Fig. 5H, right). When normalizing cocaine infusions relative to pre-surgery, ZFP189^WT^-treated mice show significantly higher rates of cocaine self-administration at the 0.05 and 0.1 mg/kg/infusion doses, whereas all other treatment groups were not significantly different from each other (Fig. 5I). This further illustrates that ZFP189^WT^ drives increased vulnerability to cocaine reinforcement, especially at small cocaine doses.

We next investigated whether ZFP189 TFs selectively altered cocaine reinforcement or impacted reinforcement of nondrug reinforcers such as sucrose. Mice were habituated to operant chambers and trained to lever press for sucrose pellets during four sequential 30-min daily sessions at FR1. Then, mice responded for sucrose pellets in a behavioral module consisting of four sequential FR5 sessions followed by one PR session (Supplementary Fig. 7A). We utilized an FR5 and PR session to be consistent with other mouse studies on sucrose reinforcement [17]. Following baseline module completion, mice were delivered ZFP189 TFs intra-NAc. Immediately following viral delivery, mice performed another sucrose reinforcer behavioral module. Active sucrose lever responding increased in all groups after surgery and no treatment condition significantly altered response rates (Supplementary Fig. 7B, C). Additionally, no viral treatment condition significantly altered sucrose break points (Supplementary Fig. 7D, E). Overall, these data indicate that NAc expression of ZFP189 TFs did not alter sensitivity to the nondrug reinforcer sucrose.

### ZFP189 function in the NAc increases cFos expression in NAc-connected brain regions

Lastly, we sought to uncover the brain circuit-wide consequences of altered ZFP189 NAc function. Mice were virally manipulated with one of our ZFP189 TFs, treated with 10 mg/kg cocaine for seven sequential days, brain tissues were fixed by 4% paraformaldehyde via transcardial perfusion, and whole brains were subjected to SHIELD tissue clearing [18], cFos labeling, and light sheet microscopy to detect cFos+ nuclei by brain regions annotated to the Allen Brain Atlas across the whole brain. cFos is commonly used as a transcriptional marker of cellular activity, enabling us to infer the brain-wide consequence of ZFP189 transcriptional function in cocaine-exposed mice. When quantifying the number of cFos+ nuclei by area of the annotated brain region (i.e., cFos density), we discovered that mice with ZFP189^WT^ intra-NAc experienced broadly elevated cFos densities across detected regions throughout the brain (Supplementary Fig. 8A, complete raw data in Supplementary Table 3), including NAc connected regions like the frontal cortex (Supplementary Fig. 8B&C) and midbrain dopaminergic nuclei (Supplementary Fig. 8D). These cFos expression data suggest that NAc ZFP189 function promotes cellular activity in NAc-connected brain regions following cocaine exposure, and the increase in cFos density across brain regions indicates that NAc ZFP189^WT^ function recruits the activity of brain cells that would otherwise not be recruited in other viral treatment conditions.

## Discussion

Here, to illuminate the drug-specific functions of the TF ZFP189, we created synthetic ZFP189 TFs for viral delivery to mouse NAc. By inverting the natural, repressive gene regulation of ZFP189^WT^ to gene activation with ZFP189^VPR^, we discover that NAc ZFP189-mediated transcriptional control uniquely facilitates cocaine-induced molecular and behavioral adaptations. In contrast, viral delivery of synthetic ZFP189 TFs to the NAc did not affect morphine-, saline-, or food-related behaviors, suggesting that NAc ZFP189-mediated transcription acts to selectively facilitate cocaine-induced brain changes. This implicates the molecular action of ZFP189 in the NAc as uniquely causal in enabling the pathogenesis of CUD-related brain adaptations. Further, by dysregulating these NAc molecular processes with ZFP189^VPR^, we illuminate potential avenues for CUD-targeted therapies which may have the capacity to inhibit the worsening of CUD.

In this research, we discovered that *Zfp189* mRNA is expressed and translated into functional ZFP189 TFs following cocaine and morphine administration in both cell culture and in the NAc (Supplementary Figs. 1–3) [9]. However, our viral delivery of synthetic ZFP189 TFs to NAc is only capable of regulating behaviors (Figs. 2, 4, 5) and transcription (Fig. 3, Supplementary Figs. 5, 6) in mice that have been exposed to cocaine. There are multiple possibilities for why the functions of NAc ZFP189 appear to be cocaine-specific. Given that our in vitro luciferase experiments demonstrated that ZFP189 TFs could regulate transcription, but only in the presence of ZFP189 REs (Supplementary Fig. 4), it is possible that cocaine exposure induces a specific chromatin state in the nuclei of transduced NAc neurons that preferentially exposes ZFP189 REs and makes ZFP189 target genes accessible for regulation, whereas ZFP189 REs remain inaccessible in mice that are not exposed to cocaine. In this scenario, cocaine experience renders a NAc chromatin landscape amenable to ZFP189 function. Alternatively, it is conceivable that cocaine and morphine induce ZFP189 expression and function in distinct NAc cell types which are not equivalently targeted by our viral method for delivering synthetic ZFP189 TFs. The interplay between ZFP189 function and drug-induced epigenetic status and the NAc cell-type specific expression patterns of ZFP189 are important future directions of this work to more clearly delineate the molecular origin of ZFP189’s cocaine-specific function.

Further, we observe that blocking dopamine or opioid receptors is not sufficient to prevent cocaine- or morphine-driven increases in *Zfp189* expression and function in cultured N2a cells (Supplementary Fig. 2). This suggests that cocaine and morphine are not exclusively producing drug dose-dependent increases in ZFP189 function via altered signaling through dopamine or opioid receptors. It is possible that cocaine and morphine are affecting ZFP189 function via pharmacological action at other receptors, such as sodium channels [19]. Alternatively, there is a strong and growing body of work to suggest that KZFPs like ZFP189 bind and repress the replication of genomic transposable elements (TEs) [13, 15, 18]. It is possible that the presence cocaine or morphine in either cultured N2a cells or the NAc is detected via molecular mechanisms partially independent from neurotransmitter receptor signaling, promotes TE release, and activates the expression and function of KZFPs like ZFP189 in order to re-stabilize these drug-released TEs. In support of this premise, we and others have identified that psychostimulant exposure robustly activates the release of TEs, including in NAc [20–23]. Since TEs are important regulators of chromatin state [24, 25], the degree to which other addictive drugs, like opioids, release NAc TEs and how these TE dynamics differ by drug class may begin to explain drug-specific epigenetic states and the cocaine-specific action of ZFP189 we observe here. This is an important future direction. While we anticipate that a portion of the direct ZFP189 gene targets are indeed TEs [15], the collective transcriptional consequence of ZFP189 function is varied, owing to non-TE ZFP189 gene targets [13, 26] and the *cis-*regulatory control of TEs on canonical genes [27]. Thus, in this work, we are studying the collective transcriptional consequence of NAc ZFP189 function (ZFP189^WT^) or dysfunction (ZFP189^VPR^) to understand the lasting molecular consequence of ZFP189 transcriptional control in distinct drug contexts downstream of the immediate molecular action of this TF. Nevertheless, the temporal mechanistic resolution of direct ZFP189 gene targets is an important future research area.

Importantly, by utilizing synthetic ZFP189 TFs in this work, we are able to augment the progression of cocaine-induced behavioral and molecular adaptations. These ZFP189-initiated behavioral changes last beyond the *trans*-gene expression timeline of HSVs (Fig. 5), which is 7–10 days post viral surgery [16, 28]. We interpret these lasting behavioral consequences to mean that NAc ZFP189 function initiates gene transcription that produces lasting changes in brain function, which is not dependent on the sustained presence of ZFP189 itself.

We interpret the collective results of this research to indicate that ZFP189^WT^ casually facilitates the molecular processes that produce cocaine-induced brain changes. We discover that ZFP189^WT^ increases the density of cFos+ cells throughout the brain and in NAc-connected brain regions (Supplementary Fig. 8), ZFP189^WT^ reduces cocaine-induced locomotor responses and cocaine CPP (Figs. 2 and 4), and ZFP189^WT^ potentiates and accelerates an escalation in cocaine IVSA behaviors, even following periods of abstinence (Fig. 5C, D), and ZFP189^WT^ sensitizes an animal to self-administer small cocaine doses (Fig. 5H, I). The IVSA cocaine dose-response data especially suggest that heighted NAc ZFP189 function, achieved by viral delivery of ZFP189^WT^, sensitizes animals to cocaine reinforcement. Vertical shifts on the ascending limb of the dose-effect function indicate higher drug reinforcer efficacy and are interpreted as increased vulnerability to drug addiction [29, 30]. This enhanced sensitivity to the reinforcing efficacy of small cocaine doses would facilitate the progression to cocaine addiction and maladaptive behavioral allocation towards cocaine at the expense of more adaptative nondrug reinforcers [31].

In ZFP189^VPR^-treated mice, rates of cocaine self-administration were lower following viral infusion compared to GFP and ZFP189^WT^-treated mice (Fig. 5C, D). ZFP189^VPR^ produced a brain transcriptional state dissimilar to the canonical transcriptional response to cocaine (Fig. 3D), and downregulated genes associated with synapse formation and maintenance (Fig. 3C). Thus, the synthetic transcriptional control of ZFP189^VPR^ may have functioned to counteract the natural cocaine-induced transcriptional adaptations and synaptic plasticity. Further, in our IPA analysis, the NMDA antagonist APV was the top predicted upstream activator of ZFP189^VPR^-driven DEGs (Fig. 3D). Interestingly, APV micro-infusion into the NAc of rats has previously been shown to selectively modulate cocaine IVSA without affecting heroin IVSA [32], further supporting the idea that these brain molecular processes are specifically involved in regulating behavioral responses to cocaine. Moreover, treatment with the NMDA antagonist ketamine, but not memantine, decreases cocaine-taking behaviors in humans [33–36]. Thus, pharmacological strategies to induce a gene expression profile akin to ZFP189^VPR^ treatment, such as APV, may hold promise as CUD-specific treatments to counteract the brain molecular adaptations to cocaine use.

Distinct work from our group and others has identified heightened *Zfp189* expression and function in the PFC as driving resilience to stress-induced social deficits [11]. More recently, we discovered that viral delivery of these same synthetic ZFP189 TFs to the neurons of the PFC alter social behaviors, with particular impact on the social cognition necessary for participating in a social group [15]. While the function of TFs can vary by cell type and brain region [9, 11, 15, 37–39], and we have no evidence that synthetic ZFP189 TFs in the NAc affect social behaviors, it is possible that ZFP189 regulates the brain molecular mechanisms that both affect social and drug use behaviors. In support of the converging molecular origin of these two phenotypes, there is growing appreciation that drug use is anti-correlated with social connectedness, in laboratory animals and humans [40–53]. Thus, studying the ZFP189-regulated molecular mechanisms at the intersection of drug use and social behavior is an important future direction of this work.

Lastly, our earlier work utilizing CRISPR/dCas9 vectors to activate *Zfp189* expression both in whole NAc and in a medium spiny neuron (MSN)-dependent manner revealed *Zfp189* expression in the NAc *Drd2* + MSN subtype as the molecular signature of chronic cocaine use and specifically responsible for diminishing cocaine-conditioned behaviors [9]. In this work, we did not conditionally deliver the synthetic ZFP189 TFs to specific NAc cell types, so we cannot be certain of the NAc cell types most responsible for the behavioral effects seen here. Combining conditional delivery of these ZFP189 TFs with behavioral tests and single-cell transcriptomics is an important future direction for this work.

In summary, we use novel synthetic TFs to simultaneously uncover and manipulate the brain molecular processes that facilitate specific SUDs. We discover NAc ZFP189 function as uniquely contributing to CUD and reveal new molecular insights as the foundation for the design of future CUD-specific treatments.

## Methods

### Neuro-2a cell culture, transfection, and analysis

*Mus musculus* Neuro-2a (N2a; ATCC^®^ CCL-131™) neuroblast cells were grown in adherent culture with 1:1 EMEM enriched/EMEM growth medium mixture (Quality Biological, #112-039-101; ATCC, #30­ 2003 or Corning, #10-009-CV) with 5% FBS (HyClone, #SH30071.03IH30-45) and 1.5% Penicillin Streptomycin (Gibco, #15140122) in a 37 °C and 5% CO_2_ Thermo Scientific HERAcell-150i CO_2_ incubator using aseptic techniques. Annual mycoplasma testing (Invitrogen MycoFluor™ Mycoplasma Detection Kit, M7006) was negative. N2a cells were maintained by passaging twice per week. One day before transfection, N2a cells of passage 60 or fewer were seeded in a 96-well plate at ~1.5 × 10^4^ cells/well. On the following day, the plasmid DNA of our ZNF189 response element (RE) *luciferase* reporter (25 ng) was co-transfected alongside our plasmid constructs of interest (i.e., CRISPR construct or synthetic ZFP189 TF) (100 ng or equal molar weight) on 80% confluent wells in triplicate for all treatment conditions with QIAGEN Effectene Transfection Kit (#301427) according to manufacturer instructions. Each reaction was carried out in 10 µl of buffer EC, 0.3 µl of Enhancer and 1 µl of Effectene, that was diluted to 100 µl in the medium before applying to the cells. The corresponding empty reporter vector RL (LightSwitch, #S990005), which lacks ZNP189 REs (here called ZFP189 Null), and control expression vector GFP were used as background controls. The plates were centrifuged for 7 min at 2000 rpm for higher transfection efficiency and were incubated for 1–3 days by covering with a Breathe-Easy sealing membrane only (Sigma-Aldrich, # Z380059-1PAK). All assays were performed 1–3 days following transfection. In experiments, each *n* corresponds to a distinct well, and experiments were replicated across 2–3 transfection reactions performed on different days. For experiments in Supplemental Fig. 2, compounds were applied in the culture media 1.5 h before experimentation.

#### Western blot

Adherent cells were washed with ice-cold dPBS and lysed in 300 μL of a 1:1 mixture of Pierce™ RIPA Buffer (Thermo Scientific, #89900) and 2x Laemmli (BioRad#1610737) on ice for 10 min. Cells were scraped and further lysed with 1.4 mm ceramic beads (Qiagen #13113) using TissueLyser LT (Qiagen # 85600) for 1 min at 50 Hz. Tubes were centrifuged at 14,000 rpm for 15 min at 4 °C and supernantant was recovered. Protein concentration was quantified using Pierce™ 660 nm Protein Assay Reagent (Thermo Scientific, #22660). Western blot was performed. 5 μl of β-mercaptoethanol (Sigma-Aldrich # M6250) was added to 100 μl cell lysate and heated to 95 °C for 5 min. 15 μg of protein was loaded to a 10% Criterion™ TGX Stain-Free™ 18-well Protein Gel (BioRad, #5678034), and separated via electrophoresis at 100 V for 2 h. Total protein was transferred to LF PVDF membrane using Trans-Blot Turbo RTA Midi 0.45 µm LF PVDF Transfer Kit (BioRad, #1704275) in Trans-Blot® Turbo™ Transfer System (BioRad #1704150) at Standard SD program (25 V constant, up to 1.0 A, for 30 min). The membranes were blocked for 1 h in 5% non-fat milk (BioRad, #1706404) at room temperature and incubated overnight at 4–7 °C with 1:5000 rabbit anti-ZFP189 (Sigma ABE2917-100UL) in 2.5%BSA (Sigma #A9647). Membranes were washed 3x in TBST (BioRad, BUF028) for 5 min. The membranes were then incubated for 1 h at room temperature with three antibodies of 1:5000 StarBright Blue700 anti-Rabbit + 1:7500 hFAB™Rhodamine Anti-Tubulin + 1:5000 hFAB™Rhodamine Anti-Actin in 15 ml TBST (BioRad, #12004161, 12004165, 12004163). Membranes were washed 6x in TBST (BioRad, BUF028) for 5 min. All the images were obtained using BioRad ChemiDoc™ MP Imaging System (BioRad #12003154), and the band intensity was read using BioRad Image Lab software. The ZFP189 band intensity was normalized by tubulin and β-actin.

#### Quantitative real-time PCR

Total RNA was extracted with Qiagen RNeasy Mini Kit protocol with a DNase (Qiagen# 79254) digest step by QIAcube Connect MDx (Qiagen# 9003070). RNA concentration was measured using Nanodrop One^C^ (ThermoFisher) and all RNA was brought to 100 ng/μL. RNA was converted to cDNA (iScript cDNA Synthesis Kit, BioRad 1708890) and the qPCR reaction was performed using Power Up SYBR Master Mix (Applied Biosystems™ #A25742/25777) and in QuantStudio 7pro (Thermo Fisher) with the following primers: GAPDH (F: AGGTCGGTGTGAACGGATTTG, R: TGTAGACCATGTAGTTGAGGTCA), β-Actin (F: TGTTACCAACTGGGACGACA, R: GGGGTGTTGAAGGTCTCAAA), ZFP189 (F: CATCAGAGC CCCAATGTCGT, R: TAACCCCACTCCTCCTTTGG), HPRT1 (F: GGTCCTTTTCACCAGCAAGCT, R: GCAGTACAGCCCCAAAATGG), and 18S rRNA (F: CCTGGATACCGCAGCTAGGA, R: GCGGCGCAATACGAATGCCCC).

#### Luciferase assay

To assess the cell viability and toxicity, on day 2 following GFP reading, we conducted a Luciferase Assay Report using the CellTiter-Glo 2.0 Assay (Promega #G9242) and Renilla luciferase assay system (Promega #E2820). Relative Luminesce Unit (RLU) was measured by the BMG-Omega plate reader using the Luminescence endpoint program with gain 3432.

### Animals

C57BL/6J male and female mice, aged 8–12 weeks, were acquired from The Jackson Laboratory. Animals were group housed (5 mice/cage; except IVSA mice which were 1 mouse/cage), at 22–25 °C in a 12-h light–dark cycle and provided food and water *ad libitum*. All tests were conducted during the light cycle. All animal procedures were performed in accordance with guidelines of the Institutional Animal Care and Use Committee at the Virginia Commonwealth University School of Medicine under the approval number AD10002174.

### Viral packaging

We de novo synthesized ZFP189^NFD^, ZFP189^WT^, and ZFP189^VPR^ and sub-cloned into HSV expression plasmids via ThermoFisher Scientific gateway LR Clonase II cloning reaction and Gateway LR Clonase II Enzyme mix kit (catalog number 11791-020 and 11971-100). Colonies were Maxiprepped (Qiagen Cat # 12163) and shipped to the Gene Delivery Technology Core at Massachusetts General Hospital for HSV packaging. Once packaged, aliquots were made and stored in –80 °C to be used in viral gene transfer through stereotaxic surgery.

### Stereotaxic infusions

Stereotaxic surgeries targeting the NAc were performed as previously [9, 11, 53]. Mice were anesthetized with an I.P. injection of ketamine (100 mg/kg) and xylazine (10 mg/kg) dissolved in sterile saline solution. Subsequently, mice were placed in a small-animal stereotaxic device (Kopf Instruments) and the skull surface was exposed. 33-gauge needles (Hamilton) were utilized to infuse 1.0 μL of virus at a rate of 0.2 μL/min followed by a 5-min rest period to prevent backflow. The following coordinates were utilized for Nac (from Bregma: anterior/posterior +1.62 mm, medial/lateral +1.5 mm, dorsal/ventral −4.4 mm; 10° angle) [9, 53–55]. These coordinates and viral volume transduce both the core and shell of the mouse NAc. As such, we are not able to ascertain the singular contributions of NAc sub-structures in these studies and all studies of the NAc here include both the core and shell. All procedures occurred in our biosafety level (BSL) 2+ facility. Mice were randomly assigned to viral treatment groups. In the course of experimentation, mice were given neutral identifiers to pseudo-blind experimenters to their viral treatment until the completion of the experiment.

### Tissue collection

Cervical dislocation was performed without anesthesia before mice were rapidly decapitated, brains were sectioned into 1 mm coronal slices using ice-cold brain matrices (Zivic Instruments: Pittsburgh, PA), and bilateral tissue punches were collected from the nucleus accumbens (NAc; 2 × 14 gauge; internal diameter 1.6 mm). Tissue was frozen on dry ice and stored at –80 °C, as is the procedure for our group [9, 15, 22, 56, 57].

#### Behavioral paradigms

##### Locomotion assay

Locomotion analysis consisted of mice being injected with a substance and then placed in an open field chamber box to move freely for an allotted time, with the distance recorded. Locomotor assays were performed for following either cocaine (10 mg/kg), morphine (10 mg/kg), or saline injections. Injections were intraperitoneally (I.P.) for cocaine and saline, and subcutaneous (S.C.) for morphine. Locomotion was captured via Ethovision tracking software as total distance moved in centimeters. Cocaine and saline condition trials were 30 min, whereas morphine trials lasted 60 min, to account for differing drug pharmacodynamics. Trials took place once per day (at mid-day) for seven days.

### Conditioned place preference (CPP)

For CPP, mice were placed in a three-chambered CPP box with time spent between the three chambers being recorded via Ethovision, in line with previously published work [9]. CPP procedures began with a pretest where mice were placed in the CPP box and had access to all three chambers for 20 min to assess baseline preferences. Viral ZFP189 TF treatment groups were balanced according to the pretest preferences to avoid bias for the chambers. Pairing chambers to a drug (cocaine or morphine) or saline was dependent upon these pretest preferences with the goal for the mice to receive drug in the less preferred chamber. Mice would be surgically manipulated the day following the pretest, and then be given a single recovery day. For the next two days, mice were conditioned by injections in both the morning (saline) and evening (drug) and then restricted to one chamber of the box for 30 min. Following the conditioning phase mice underwent a post-test during which, mice were placed in the CPP box with free access to all chambers of the box for a 20 min test session. Data are represented as time spent in the drug-paired chamber posttest minus pretest, to determine the amount of drug-paired change which occurred.

### Self-administration procedures

#### Jugular vein catheterization

Mice were prepared for the surgery first by removing fur at the surgical site with electric clippers, then the skin at the surgical site was aseptically prepared with alternating applications of 70% ethanol and chlorhexidine solution. The mouse was then moved to a laminar flow bench, placed in dorsal recumbency. An incision was made over the right jugular vein, until the vein was exposed. Once exposed a second small incision was made in the vein for the catheter to be inserted into, then secured by a non-absorbable suture. Next an incision was made in the intrascapular region, for the free end of the catheter to be subcutaneously tunneled and exteriorized. The catheters were then secured to the vascular access button (VAB), and then filled with a heparin/glycerol lock solution. Finally, a silicone mesh disk portion of the VAB is placed subcutaneously. Local anesthetics were then applied topically to incision sites and closed using non-absorbable sutures.

#### Jugular vein catheter maintenance

Catheters were maintained through a routine of sterilization that involved wiping the VAB with ethanol-soaked cotton swabs before and after being placed in IVSA chambers, and flushed daily with a 0.05 ml heparinized saline (30 U) containing ampicillin antibiotic (5 mg/ml) via a pinport tipped syringe. During periods of forced abstinence, a glycerin-based locking solution was used to fill the catheters, with 0.01 mL being put into the VAB. Mice could remain locked for a maximum of 7-days before requiring being unlocked and flushed with the heparinized saline solution described above. To unlock the VAB, this locking solution was drawn up via an empty pinport tipped syringe until a small amount of blood was drawn, to indicate patency.

#### Anesthetization and surgery of catheterized mice

Intra-NAc infusion of ZFP189 TFs via stereotaxic surgery was consistent with the procedure described above in the Stereotaxic Infusion section, with one additional step. To avoid damaging the jugular vein catheters, mice were briefly anesthetized (5–10 s) using a bell jar with isoflurane to allow for the I.P. injection of the ketamine and xylazine solution without needing to scruff the animals. The rest of the surgical procedure proceeded as above.

#### Operant chambers

The operant chambers used in this study were from Med-associates, classic modular test chambers (ENV-307A-CT), placed in Standard MDF sound attenuating cubicles (ENV-022MD). The operant chambers contained a LED house light (ENV-315M-LED) that would be on during sessions, two retractable levers (ENV-312-3M) along with two cue lights to indicate active and inactive levers (ENV-321M). The operant chamber was also paired with Pellet dispenser (ENV-203-20) and pellet receptacle (ENV-303M) which allowed for the administration of sucrose pellets in food training/testing. The set-up also included a variable speed syringe pump (PHM-210) that allowed for the infusion of cocaine via tubing to the catheters implanted in the mice.

#### Cocaine infusions via syringe pump

To deliver 0.5 mg/kg cocaine per IV infusion, syringes were filled with 0.25 mg/ml of cocaine in saline, which would be delivered with the med-associates syringe pump running at a rate of 3.33RPM for 2.82 s. This rate was calculated based on the manufacturer’s recommendation. For the dose response experiments the pump remained at the 3.33RPM rate for 2.82 s, for all doses. To deliver 0.05 mg/kg/infusion, syringes were filled with 0.025 mg/ml of cocaine in saline, to deliver 0.1 mg/kg/infusion, syringes were filled with 0.05 mg/ml of cocaine in saline, and to deliver 1.0 mg/kg/infusion, syringes were filled with 0.5 mg/ml of cocaine in saline.

#### Training-cocaine IVSA fixed ratio 1 scheduled study

Mice were trained on cocaine training sessions lasting 3-h and on a FR1 reinforcement schedule. The active lever was indicated via a stim-light illuminated above, which would turn off during the timeout period. Active lever presses during timeouts were recorded but did not result in infusions. Nor did inactive lever presses throughout the entire session. During the first two sessions cocaine infusions were capped at a maximum of 60, with a timeout period of 15-s following each infusion to avoid the potential for overdose. At the start of the third day, the cap cocaine on infusions was elevated to 120 per session, but the timeout period remained unchanged. Mice remained in this training until they showed an ability to discriminate between active and inactive levers and a stable response rate was achieved, defined as individual animal infusions in the last five sessions that did not differ by more than 35% from the average of the last two sessions, inclusive, or the group average did not vary by more than 20% over the last two sessions, inclusive. Generally, this process took between 7 to 10 days. Following the achievement of stable responding rates, mice would undergo the intra-NAc infusion of ZFP189 TFs.

#### Training-sucrose reinforcement study (non-catheterized mice)

For the sucrose self-administration experiment, mice were introduced to unflavored sucrose pellets from Bioserv (F07595) while in their home cages, to avoid neophobia. Training consisted of four daily 30-min sessions, under a Fixed-Ratio 1 schedule of reinforcement. Mice that reached a stable response rate (defined as the number of sucrose pellets earned did not differ within a subject by more than 35% from the average of the last two sessions, inclusive, or the group average did not vary by more than 20% over the last two sessions, inclusive, or earning a minimum of 20+ sucrose pellets in two consecutive sessions) progressed to the behavioral module described above.

#### Module-based behavioral testing system

Fixed-Ratio 5 (FR5) and Progressive-Ratio (PR) schedules of reinforcement (described below) were used to rigorously understand how ZFP189 TFs impact both reinforcement-seeking and motivation-based behaviors. An FR5 schedule of reinforcement (i.e., five consecutive active lever responses are required for reinforcer presentation) was utilized to rigorously assess ZFP189 TFs on cocaine reinforcement and minimize the likelihood that mice would receive a cocaine infusion through non-deliberate interactions with the lever (i.e., bumping into the lever while moving around the chamber). The PR schedule progression was based on the following equation Response Ratio (rounded to the nearest integer) = [5e^(injection number X 0.2)^]–5, in line with Richardson and Roberts, 1996 [58]. Statistical analysis for PR was performed measuring the reinforcers earned in sessions. Behavioral modules were structured with four FR5 sessions followed by three PR sessions. The module structure remained consistent prior to surgery (M1), post-TF infusion (M2), and following FA (M3).

#### Dose response IVSA

Acquisition occurred using the same FR1 training system as used for the module cocaine testing. Dose response (DR) testing sessions were performed on a FR5 reinforcement schedule and lasted 3-h, in line with the module based FR5 testing. Four doses were used in this study; 0.05, 0.1, 0.5, 1.0 mg/kg per cocaine infusion. Each animal was tested at every dose for 4-consecutive sessions at each dose, with the order in which mice went through the doses was scrambled per individual mouse. Four sessions occurred at each dose with 4-doses, meant each DR curve required 16 consecutive testing sessions. Mice were tested prior to surgery to establish a baseline DR curve, and once again following the intra-NAc infusion of ZFP189 TFs.

### RNA sequencing

During tissue collection, targeting and expression were assessed using GFP as a marker for virus. Samples that did not show expression, or had off target expression were excluded from the RNAseq sample groups. Total RNA was extracted from fresh frozen tissue samples using Qiagen RNeasy Plus Universal mini kit following manufacturer’s instructions (Qiagen, Hilden, Germany). RNA samples were quantified using Qubit 2.0 Fluorometer (Life Technologies, Carlsbad, CA, USA) and RNA integrity was measured using the RNA Screen Tape on Agilent 2200 TapeStation (Agilent Technologies, Palo Alto, CA, USA). The average RNA integrity number for all samples exceeded 9.1. Samples were initially treated with TURBO DNase (Thermo Fisher Scientific, Waltham, MA, USA) to remove DNA contaminants. The next steps included performing rRNA depletion using QIAseq® FastSelectTM−rRNA HMR kit (Qiagen, Germantown, MD, USA), which was conducted following the manufacturer’s protocol. RNA sequencing libraries were constructed with the NEBNext Ultra II RNA Library Preparation Kit for Illumina by following the manufacturer’s recommendations. Briefly, enriched RNAs are fragmented for 15 min at 94 °C. First strand and second strand cDNA are subsequently synthesized. cDNA fragments are end repaired and adenylated at 3′ends, and universal adapters are ligated to cDNA fragments, followed by index addition and library enrichment with limited cycle PCR. Sequencing libraries were validated using the Agilent Tapestation 4200 (Agilent Technologies, Palo Alto, CA, USA), and quantified using Qubit 2.0 Fluorometer (ThermoFisher Scientific, Waltham, MA, USA) as well as by quantitative PCR (KAPA Biosystems, Wilmington, MA, USA). The sequencing libraries were multiplexed and clustered on one lane of a flowcell. After clustering, the flowcell was loaded on the Illumina HiSeq 4000 instrument according to the manufacturer’s instructions. The samples were sequenced using a 2 × 150 Pair-End (PE) configuration at a sequencing depth of ~37 million reads per sample (mean = 37 M, SEM = 0.66 M). The average percent of total mapped reads for our samples was ~97% (mean = 96.7%, SEM = 0.1%). Unique mapped reads averaged around 34 million (mean = 33.9 M, SEM = 0.61 M), with a mapped read percent at 92% (mean = 91.9%, SEM = 0.17%). Other overall sample sequencing statistics include the mean quality score (mean = 35.72, SEM = 0.008) and the percent of bases ≥30 (mean = 92.56, SEM = 0.04). Sequencing files have been submitted to GEO Omnibus and will be released upon publication.

Sequence reads were trimmed to remove possible adapter sequences and nucleotides with poor quality using Trimmomatic v.0.36. The trimmed reads were mapped to the Mus musculus GRCm38 reference genome available on Ensembl using the STAR aligner v.2.5.2b. Unique gene hit counts were calculated by using featureCounts from the Subread package v.1.5.2. After extraction of gene hit counts, the gene hit counts table was used for downstream differential expression analysis. Using DESeq2, a comparison of gene expression between groups of samples was performed. The Wald test was used to generate *p*-values and log2 fold changes. Genes with an adjusted *p*-value < 0.05 and absolute log_2_ fold change >1 were called as differentially expressed genes for each comparison. Volcano plots with these DEGs were assembled using Graphpad Prism 9.

To assess the function of the significant DEGs regulated by ZFP189 TFs, we performed a Gene Set enrichment analysis (GSEA) [59] and Ingenuity pathway analysis (IPA) [17]. For GSEA data was analyzed using WebGestalt (https://www.webgestalt.org/#) and for the IPA, the networks were generated through the use of Qiagen IPA (QIAGEN Inc., https://digitalinsights.qiagen.com/IPA). Full differential expression tables generated from DEseq2 were used in GSEA without significance thresholds. Significant DEGs from the cocaine ZFP189^VPR^ condition were analyzed using Ingenuity Pathway Analysis to determine upstream regulators. Significantly activated and inhibited upstream regulators (FDR < 0.05) were sorted by activation z-score and graphed into an activation heatmap using the ggplot package in R-studio.

#### Whole brain clearing and cFos staining

Following seven days of 10 mg/kg cocaine daily injections, mice were anesthetized with ketamine (100 mg/kg) and xylazine (10 mg/kg) then perfused. Transcardial perfusion of mice was performed with ice-cold 1X PBS with 10 μ/mL heparin until fluid ran clear, followed by ice-cold 4% PFA. Whole brains were then removed and placed in 4% paraformaldehyde (PFA) solution to incubate at 4°C for 24 h with gentle shaking. Next, these samples were washed twice in PBS and then stored in PBS with 0.02% sodium azide. PFA-fixed samples were preserved with using SHIELD reagents (LifeCanvasTech, Cambridge, MA) using the manufacturer’s instructions [18]. Samples were delipidated using LifeCanvas Technologies Clear+ delipidation reagents. Following delipidation samples were labeled using eFLASH [60] technology which integrates stochastic electrotranspor [61] and SWITCH [62, 63] using a SmartBatch+ (or SmartLabel) device (LifeCanvasTech, Cambridge, MA). After immunolabeling, samples were incubated in 50% EasyIndex (RI = 1.52, LifeCanvas Technologies) overnight at 37 °C followed by one day incubation in 100% EasyIndex for refractive index matching. After index matching the samples were imaged using a SmartSPIM axially-swept light sheet microscope using a 3.6x (0.2 NA) (LifeCanvasTech, Cambridge, MA). Automated cell detection was performed by LifeCanvas Technologies using a custom convolutional neural network created with the Tensorflow python package (Google). The cell detection was performed by two networks in sequence. First, a fully-convolutional detection network (https://arxiv.org/abs/1605.06211v1) based on a U-Net architecture (https://arxiv.org/abs/1505.04597v1) was used to find possible positive locations. Second, a convolutional network using a ResNet architecture (https://arxiv.org/abs/1512.03385v1) was used to classify each location as positive or negative. Using the previously-calculated Atlas Registration, each cell location was projected onto the Allen Brain Atlas in order to count the number of cells for each atlas-defined region. Samples were registered to the Allen Brain Atlas (Allen Institute: https://portal.brain-map.org/) using an automated process (alignment performed by LifeCanvas Technologies). These samples were imaged with SmartSPIM at 4 μm z-step and 1.8 μm xy pixel size, with the following channels: 488 nm - anti-GFP, 561 nm - NeuN, 647 nm - cFOS.

### Statistics

Additional details on the statistical test performed in each experiment can be found in Supplemental Table 4. Experimental sample sizes were based on prior experiences with each method and aligned to the common sample sizes for the field.

## Supplementary information

Supplementary Figures and Legends

Supplementary Table 1

Supplementary Table 2

Supplementary Table 3

Supplementary Table 4

## Data Availability

Raw and processed RNAseq gene expression data are available via Gene Expression Omnibus server (accession number GSE276453). Other data that supports the findings from this study are available from the corresponding author upon request.
