# Supplementary material for: The function of the ZFP189 transcription factor in the nucleus accumbens facilitates cocaine-specific transcriptional and behavioral adaptations
Source: Mol Psychiatry. 2024 Nov 25;30(6):2490–503. doi: 10.1038/s41380-024-02852-7 (PMC12092205; doi:10.1038/s41380-024-02852-7)
Supplement: Supplementary file 1 — Supplementary Figures and Legends [file 41380_2024_2852_MOESM1_ESM.docx]

**SUPPLEMENTARY FIGURES**

**The function of the ZFP189 transcription factor in the nucleus accumbens facilitates cocaine-specific transcriptional and behavioral adaptations**

Joseph A. Picone^1^, Annalise Hassan^2^, R. Kijoon Kim^1^, Diego Piñeiro Lira^1^, Gabriella M. Silva^2^, Natalie L. Truby^1^, Hadessah Johnson^1^, Collin D. Teague^3^, Rachael L. Neve^4^, Matthew L. Banks^2^, Xiaohong Cui^1^, Peter J. Hamilton^1,^*

^1^Department of Anatomy and Neurobiology, Virginia Commonwealth University School of Medicine, Richmond, VA, USA

^2^Department of Pharmacology and Toxicology, Virginia Commonwealth University School of Medicine, Richmond, VA, USA

^3^Nash Family Department of Neuroscience and Friedman Brain Institute, Icahn School of Medicine at Mount Sinai, New York, New York

^4^Gene Delivery Technology Core, Massachusetts General Hospital, Cambridge, MA, USA.

*correspondence: peter.hamilton@vcuhealth.org

1217 E Marshall St, Richmond, VA 23298

804-628-3003


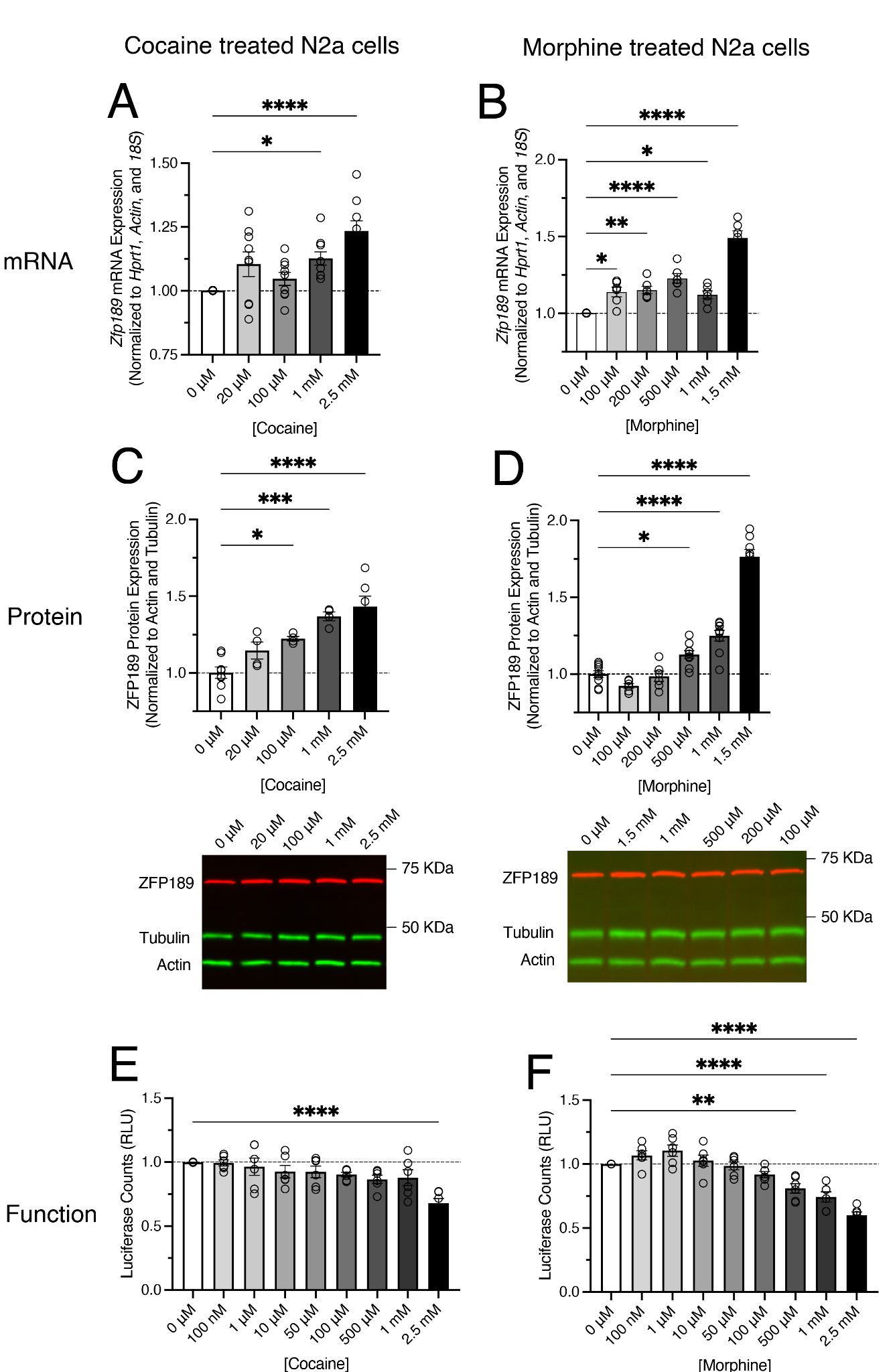


**Supplementary Figure 1: Cocaine and morphine induce the expression of functional ZFP189 transcription factor *in vitro*.** (A) *Zfp189* mRNA expression in N2a cells in response to exposure to cocaine at multiple doses. Increasing the dosage of applied cocaine induced significant increases in *Zfp189* mRNA expression. Ordinary one-way ANOVA relative to 0 µM condition (Dunnett Test); *p-value < 0.05, ****p-value < 0.0001, n = 9 wells per condition. (B) *Zfp189* mRNA expression in N2a cells in response to exposure to morphine at multiple doses. Increasing the dosage of applied morphine induced significant increases in *Zfp189* mRNA expression. Ordinary one-way ANOVA relative to 0 µM condition (Dunnett Test); *p-value < 0.05, **p-value < 0.01, ****p-value < 0.0001, n = 6 wells per condition. (C) ZFP189 protein expression in N2a cells in response to exposure to cocaine at multiple doses. Increasing the dosage of applied cocaine induced significant increases in ZFP189 protein expression. Ordinary one-way ANOVA relative to 0 µM condition (Dunnett Test); *p-value < 0.05, ****p-value < 0.0001, n = 4-8 wells per condition. (D) ZFP189 protein expression in N2a cells in response to exposure to morphine at multiple doses. Increasing the dosage of applied morphine induced significant increases in ZFP189 protein expression. Ordinary one-way ANOVA relative to 0 µM condition (Dunnett Test); *p-value < 0.05, ****p-value < 0.0001, n = 9 wells per condition. (E) Luciferase assay data with increasing dosages of applied cocaine to N2a cells. Increasing the dosage of applied cocaine induced significant decreases in luciferase counts. These decreases in RLUs suggest the ZFP189 TFs are functioning to induce transcriptional repression. Ordinary one-way ANOVA relative to 0 µM condition (Dunnett Test); ****p-value < 0.0001, n = 6 wells per condition. (F) Luciferase assay data with increasing dosages of applied morphine to N2a cells. Increasing the dosage of applied morphine induced significant decreases in luciferase counts. These decreases in RLUs suggest the ZFP189 TFs are functioning to induce transcriptional repression. Ordinary one-way ANOVA relative to 0 µM condition (Dunnett Test); **p-value < 0.01, ****p-value < 0.0001, n = 6 wells per condition.

**
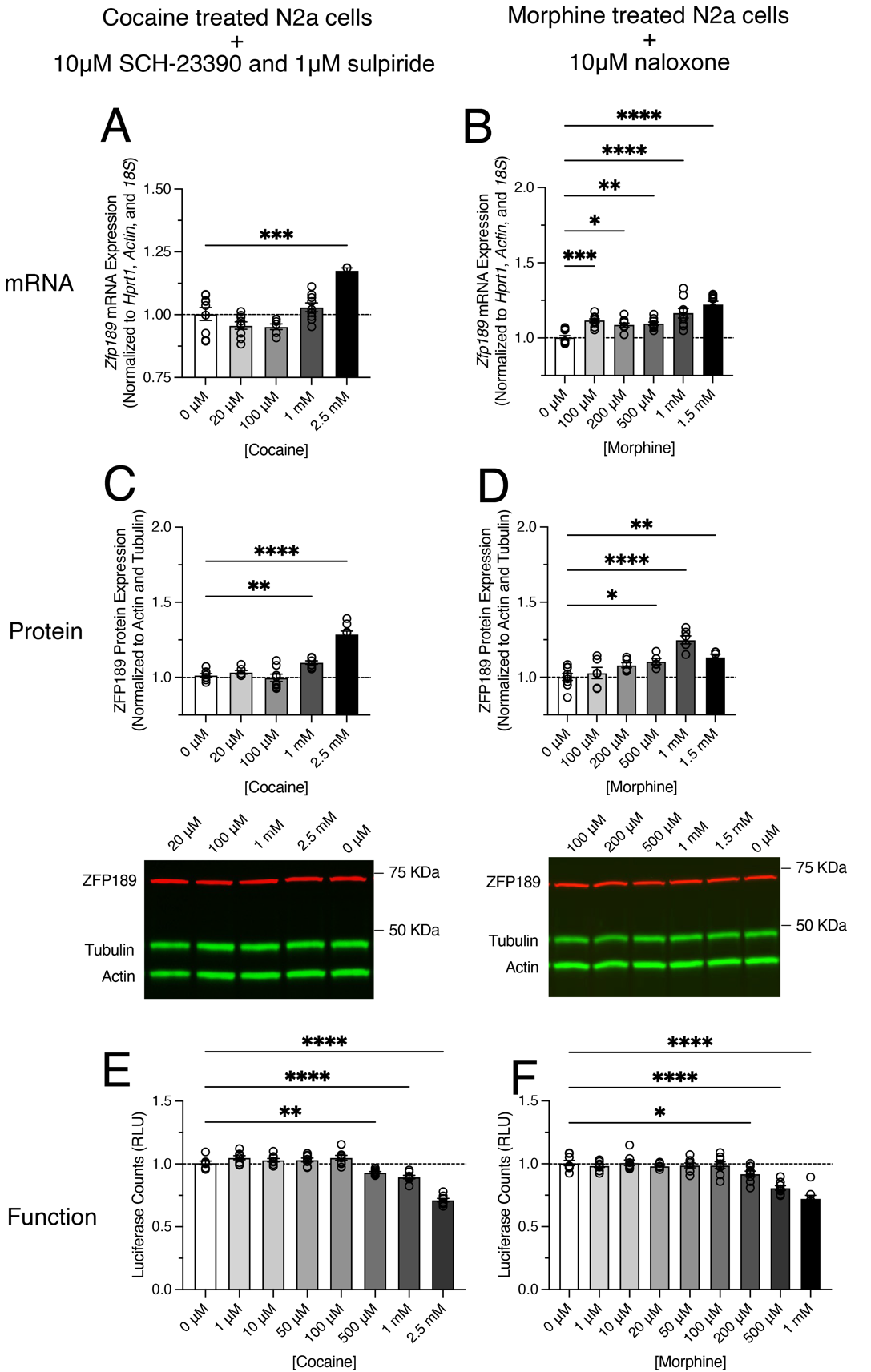
**

**Supplementary Figure 2: Blocking dopamine or opioid receptors does not prevent cocaine or morphine from activating the expression of functional ZFP189 transcription factor *in vitro*.** N2a cells were pretreated for 1.5 hours with either: 1) a combination of 10 µM SCH-23390 (a dopamine D1 receptor antagonist) and 1 µM sulpiride (a dopamine D2 receptor antagonist) and subjected to increasing concentrations of cocaine (left column), or 2) 10 µM naloxone (a non-selective opioid receptor antagonist) subjected to increasing concentrations of morphine (right column). All other experimental methods were identical to Supplementary Figure 1. (A) *Zfp189* mRNA expression in N2a cells in response to exposure to cocaine at multiple doses. Increasing the dosage of applied cocaine induced significant increases in *Zfp189* mRNA expression, even in the presence of dopamine receptor antagonists. Ordinary one-way ANOVA relative to 0 µM condition (Dunnett Test); ***p-value < 0.001, n = 9 wells per condition. (B) *Zfp189* mRNA expression in N2a cells in response to exposure to morphine at multiple doses. Increasing the dosage of applied morphine induced significant increases in *Zfp189* mRNA expression, even in the presence of opioid receptor antagonists. Ordinary one-way ANOVA relative to 0 µM condition (Dunnett Test); *p-value < 0.05, **p-value < 0.01, ***p-value < 0.001, ****p-value < 0.0001, n = 9 wells per condition. (C) ZFP189 protein expression in N2a cells in response to exposure to cocaine at multiple doses. Increasing the dosage of applied cocaine induced significant increases in ZFP189 protein expression, even in the presence of dopamine receptor antagonists. Ordinary one-way ANOVA relative to 0 µM condition (Dunnett Test); *8p-value < 0.01, ****p-value < 0.0001, n = 8 wells per condition. (D) ZFP189 protein expression in N2a cells in response to exposure to morphine at multiple doses. Increasing the dosage of applied morphine induced significant increases in ZFP189 protein expression, even in the presence of opioid receptor antagonists. Ordinary one-way ANOVA relative to 0 µM condition (Dunnett Test); *p-value < 0.05, **p-value < 0.01, ****p-value < 0.0001, n = 6-9 wells per condition. (E) Luciferase assay data with increasing dosages of applied cocaine to N2a cells. Increasing the dosage of applied cocaine induced significant decreases in luciferase counts, even in the presence of dopamine receptor antagonists. These decreases in RLUs suggest the ZFP189 TFs are functioning to induce transcriptional repression. Ordinary one-way ANOVA relative to 0 µM condition (Dunnett Test); **p-value < 0.01, ****p-value < 0.0001, n = 8 wells per condition. (F) Luciferase assay data with increasing dosages of applied morphine to N2a cells. Increasing the dosage of applied morphine induced significant decreases in luciferase counts, even in the presence of opioid receptor antagonists. These decreases in RLUs suggest the ZFP189 TFs are functioning to induce transcriptional repression. Ordinary one-way ANOVA relative to 0 µM condition (Dunnett Test); *p-value < 0.05, ****p-value < 0.0001, n = 8 wells per condition.


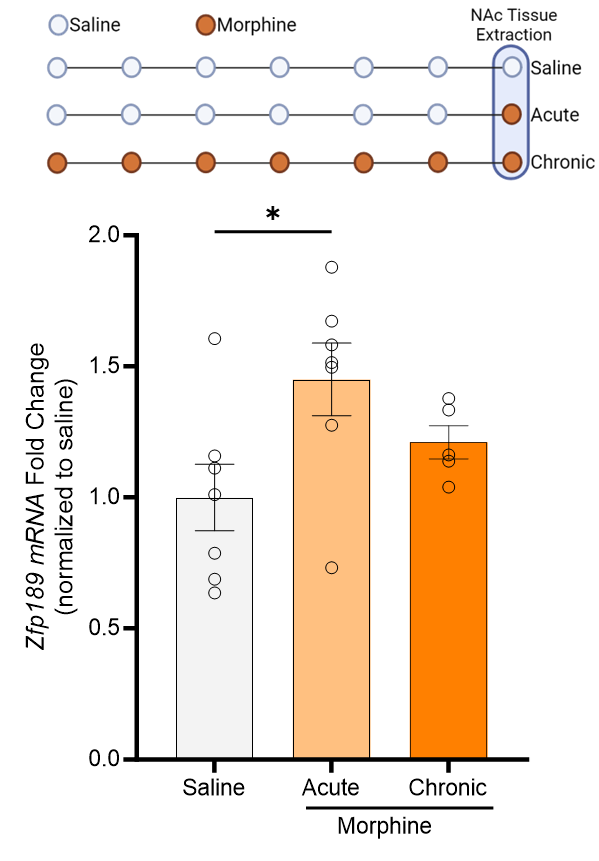


**Supplemental Figure 3: *Zfp189* expression in the nucleus accumbens is increased in response to morphine.** (A) The experimental timeline to determine the effect of acute or chronic subcutaneous injections of morphine on NAc *Zfp189* mRNA expression. Each bubble represents a day. Light blue bubbles correspond to injections of saline, whereas red bubbles correspond to injections of 10 mg/kg morphine. (B) Bilateral NAc *Zfp189* mRNA levels quantified via qRT-PCR from each treatment condition. An acute morphine injection significantly increased *Zfp189* mRNA levels relative to saline-treated animals. One way ANOVA followed by Bonferroni’s multiple comparison test; * p-value < 0.05. n = 7 (Saline; Acute morphine) or 5 (Chronic morphine).


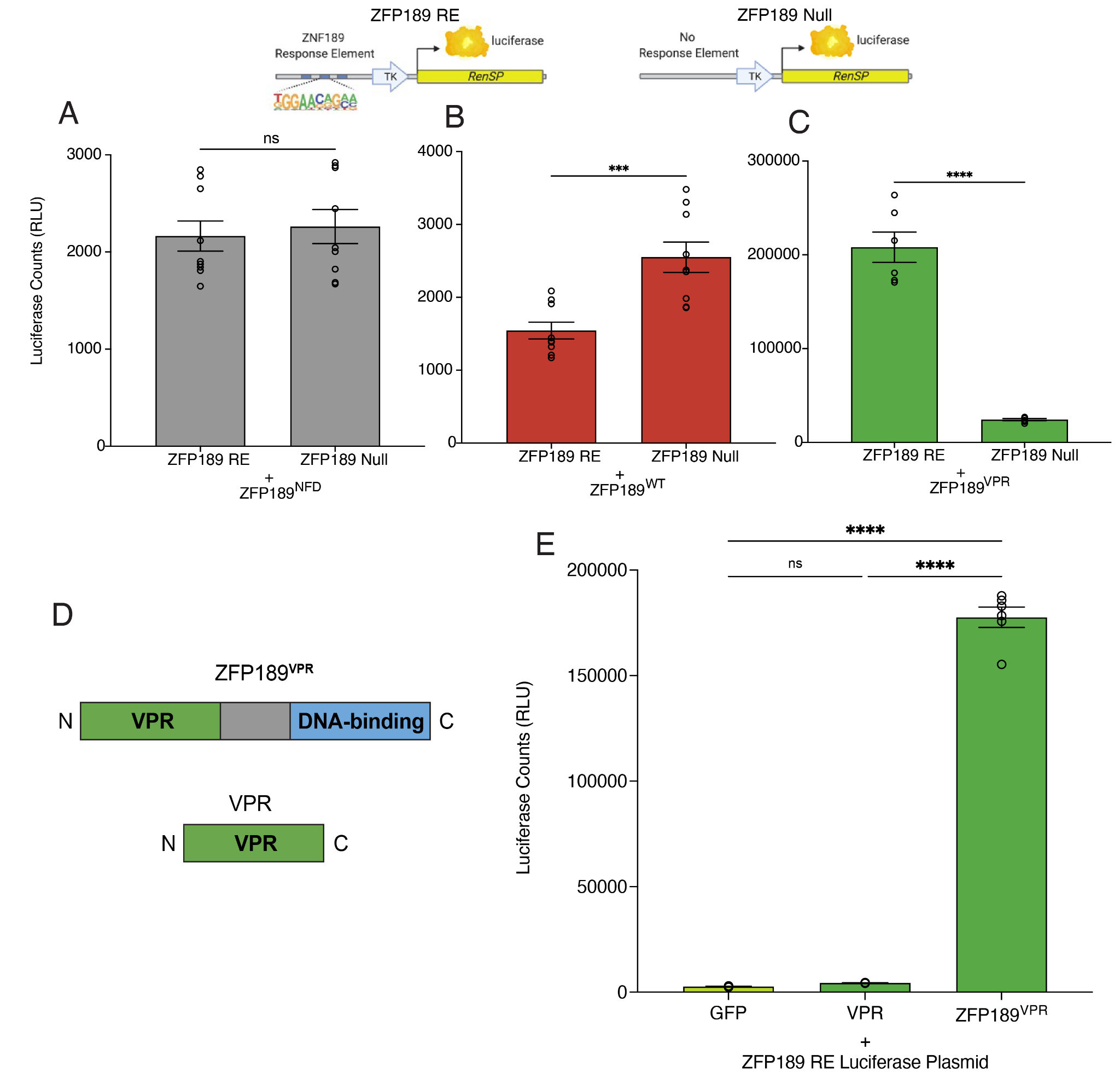


**Supplemental Figure 4: ZFP189 TFs elicit transcriptional changes only in the presence of the *Zfp189* RE with intact *Zfp189* binding domain.** (A) Removing ZFP189 REs from the promoter of our luciferase target gene (ZFP189 null) does not modify the function of ZFP189^NFD^. Two-tailed, unpaired Student’s *t*-test; ns p-value > 0.05. n = 9 per condition. (B) Removing ZFP189 REs from the promoter of our luciferase target gene releases the gene repression of ZFP189^WT^. Two-tailed, unpaired Student’s *t*-test; *** p-value < 0.001. n = 9 per condition. (C) Removing ZFP189 REs from the promoter of our luciferase target ablates the gene activating function of ZFP189^VPR^. Two-tailed, unpaired Student’s *t*-test; ****p-value > 0.0001. n = 6 per condition. (D) Graphic representation of ZFP189^VPR^ TF and VPR functional moiety by itself. (E) Luciferase counts in response to transfection with GFP, VPR, and ZFP189^VPR^. Only when the ZFP189 RE was present and ZFP189 binding domain was paired with the VPR functional domain was there a significant increase in RLU. One-way ANOVA (Tukey); ****p-value > 0.0001. n = 6 per condition.


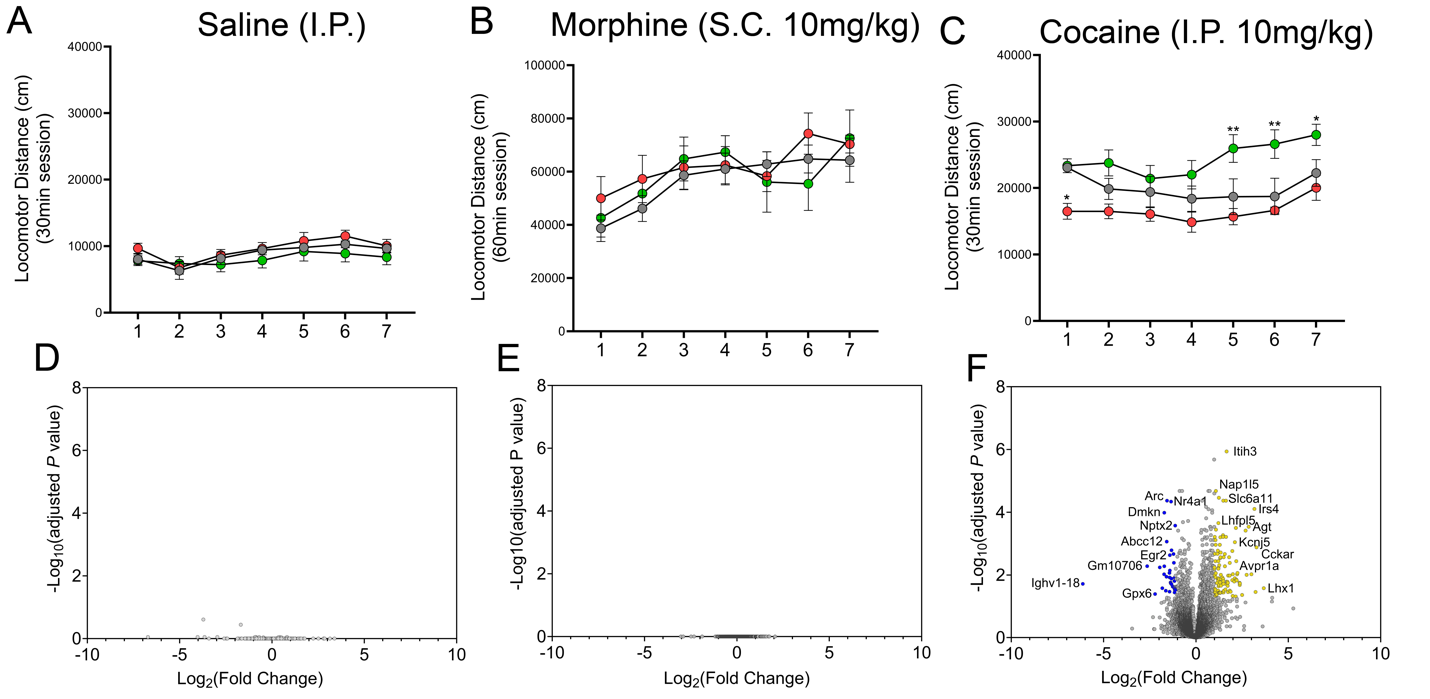


**Supplementary Figure 5: Drug-induced locomotor data for the representative mice that underwent RNA sequencing.** (A) Daily distance moved (cm) in response to saline injections by ZFP189 TF treatment condition. No significant effects observed. Two-way repeated measure ANOVA (Tukey Test). n = 5 (ZFP189^NFD^), n=5 (ZFP189^WT^) and n=5 (ZFP189^VPR^) mice. (B) Daily distance moved (cm) in response to morphine injections by ZFP189 TF treatment condition. No significant effects observed. Two-way repeated measure ANOVA (Tukey Test). n = 5 (ZFP189^NFD^), n=5 (ZFP189^WT^) and n=5 (ZFP189^VPR^). (C) Daily distance moved (cm) in response to cocaine injections by ZFP189 TF treatment condition. ZFP189^VPR^ induced increases, and ZFP189^WT^ induced significant decreases in locomotion on individual days. Two-way repeated measure ANOVA (Tukey Test); * p-value < 0.05, ** p-value < 0.01. n = 5 (ZFP189^NFD^), n=5 (ZFP189^WT^) and n=5 (ZFP189^VPR^). (D) RNAseq volcano plots for ZFP189 TFs in the NAc in context of saline. No significant DEGs appeared in the context of saline (adjusted P value >1.3 and Log2 Fold Change>1). (E) RNAseq volcano plots for ZFP189 TFs in the NAc in the context of morphine. No significant DEGs appeared in the context of morphine (adjusted P value >1.3 and Log2 Fold Change>1). (F). RNAseq volcano plots for ZFP189 TFs in the NAc in the context of cocaine. ZFP189^VPR^ induces significant DEGs relative to ZFP189^NFD^ in the context of cocaine (adjusted P value >1.3 and Log2 Fold Change>1).


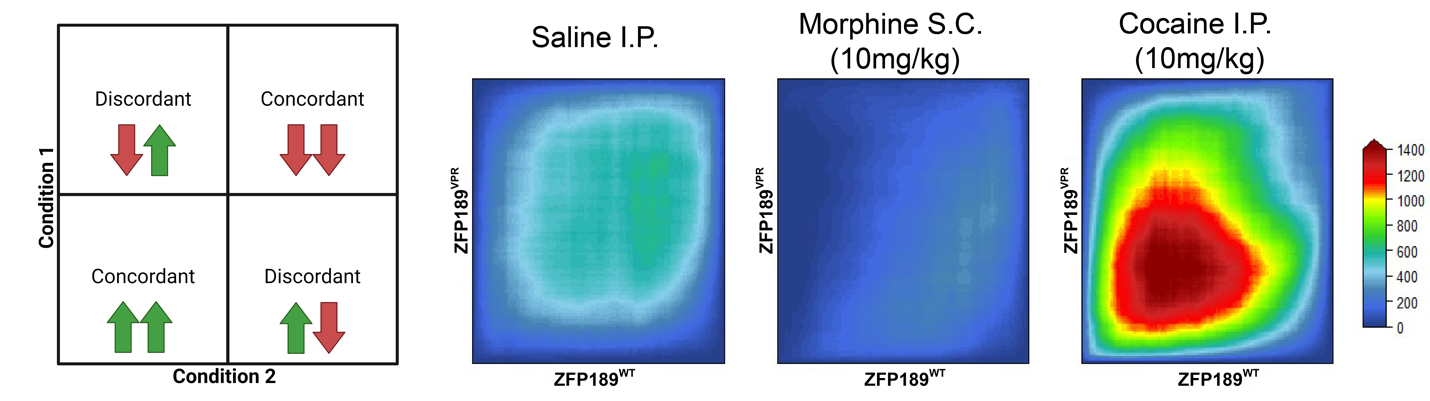


**Supplementary Figure 6: Cocaine exposure, but not saline or morphine exposure, drives robust gene expression by ZFP189^WT^ and ZFP189^VPR^.** Threshold free rank-rank hypergeometric overlap (RRHO) plots comparing all differentially expressed genes (DEGs) regulated by ZFP189^VPR^ versus ZFP189^WT^ in mice treated with saline, morphine, or cocaine. See Supplemental Table 1 for complete DEG lists for each comparison. In Left panel: Arrows represent the direction of gene expression changes across the two groups being compared (i.e., lower left quadrant represents genes up-regulated in both groups; upper right quadrant represents genes down-regulated in both groups). In Right three panels: Cocaine experience drives a strong and largely coordinated impact on nucleus accumbens gene expression between ZFP189^VPR^ and ZFP189^WT^ conditions that does not occur in mice manipulated with these same ZFP189 transcription factors and treated with saline or morphine.

**
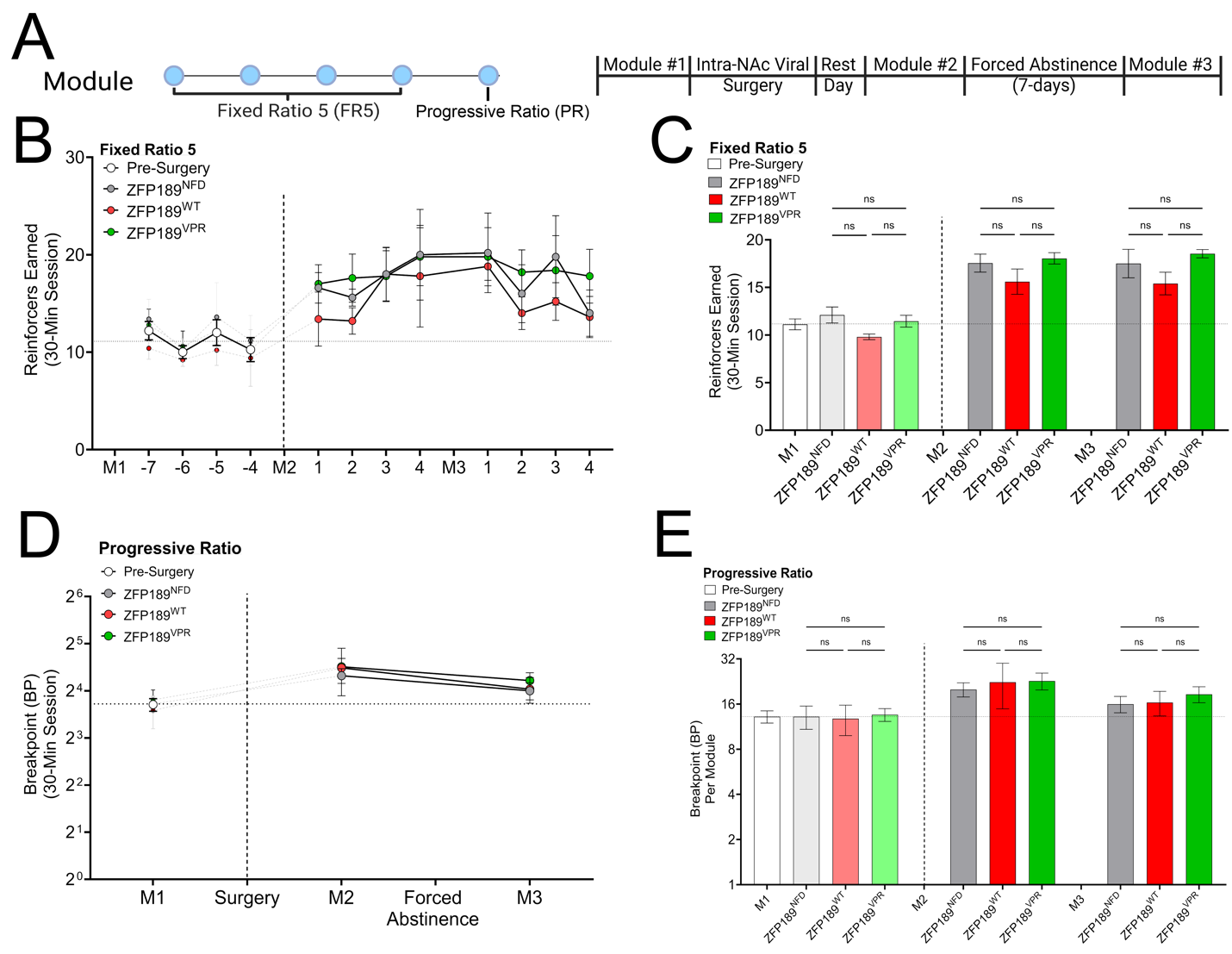
Supplementary Figure 7: ZFP189 TFs do not impact the efficacy or motivation for natural food reinforcers.** (A) Timeline and composition of operant food “module”. (B) Daily active lever presses on fixed-ratio 5 (FR5) for sucrose pellets during a 30-minute session. No significant differences were observed between any ZFP189 TFs in the first module following intra-NAc infusion, or following a FA period. Two-way ANOVA (Tukey Test); ns p-value > 0.05. n = 8 (ZFP189^NFD^), n=7 (ZFP189^WT^) and n=9 (ZFP189^VPR^). (C) Averages of active lever presses across all sessions. ZFP189 TFs do not significantly alter active lever presses for a sucrose pellet reinforcer. Ordinary one-way ANOVA (Tukey Test); ns p-value > 0.05. n = 8 (ZFP189^NFD^), n=7 (ZFP189^WT^) and n=9 (ZFP189^VPR^) mice. (D) Daily active lever presses on Progressive-Ratio (PR) for sucrose pellets during 30-minute session. No significant differences were observed between any ZFP189 TFs in the first module following intra-NAc infusion, or following a FA period. Two-way ANOVA (Tukey Test); ns p-value > 0.05. n = 8 (ZFP189^NFD^), n=7 (ZFP189^WT^) and n=9 (ZFP189^VPR^). (E) Progressive ratio value (PRV) defined as number of active lever presses last yielding a food reinforcer. No significant differences were found between any of the ZFP189 TFs in the first module following intra-NAc infusion, or following a FA period, indicating that synthetic ZFP189 TFs do not alter motivation to acquire a food reinforcer. Ordinary one-way ANOVA (Tukey Test); ns p-value > 0.05. n = 8 (ZFP189^NFD^), n = 7 (ZFP189^WT^) and n = 9 (ZFP189^VPR^) mice.

**
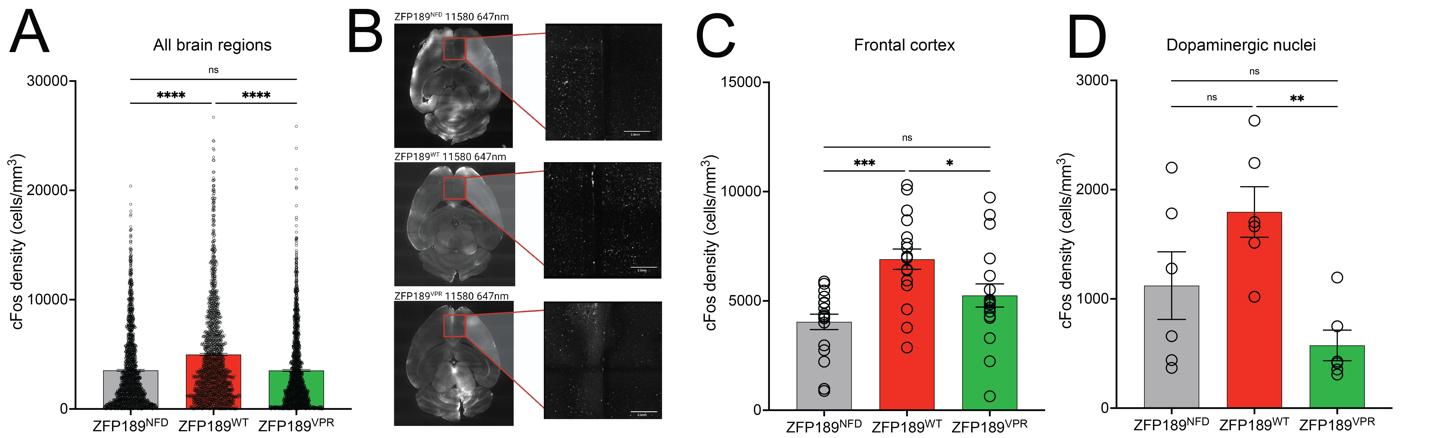
**

**Supplementary Figure 8: NAc ZFP189^WT^ increases cFos density throughout the brain and in NAc connected brain regions.** Mice were virally delivered synthetic ZFP189 TF tools within NAc and underwent seven daily 10 mg/kg injections of cocaine. Whole brains were cleared and cFos+ cells were quantified throughout the entire brain. (A) Mice expressing ZFP189^WT^ in NAc experienced increased cFos densities in annotated regions throughout the brain. Ordinary one-way ANOVA (Tukey Test); ****p-value < 0.0001, n = 3 (ZFP189^NFD^), n = 3 (ZFP189^WT^) and n = 3 (ZFP189^VPR^) mice with 1,677 distinct nuclei analyzed in each mouse. (B) Horizontal cross-sections of representative brains transduced with each of the ZFP189 TFs. cFos density was measured as cFos+ cells per mm^3^. (C) c-Fos+ density in the frontal cortex. ZFP189^WT^ showed significantly higher cFos expression density relative to ZFP189^NFD^ and ZFP189^VPR^. Ordinary one-way ANOVA (Tukey Test); *p-value < 0.05 ***p-value < 0.001, n = 3 (ZFP189^NFD^), n = 3 (ZFP189^WT^) and n = 3 (ZFP189^VPR^) mice with 18 distinct nuclei analyzed in each mouse. (D) c-Fos+ density in midbrain dopaminergic nuclei. ZFP189^WT^ showed significantly higher cFos expression density relative to ZFP189^VPR^. Ordinary one-way ANOVA (Tukey Test); *p-value < 0.05 ***p-value < 0.001, n = 3 (ZFP189^NFD^), n = 3 (ZFP189^WT^) and n = 3 (ZFP189^VPR^) mice with 6 distinct nuclei analyzed in each mouse. See Supplementary Table 3 for data from all brain regions and the selected regions to analyze frontal cortex and dopaminergic nuclei in panel C and D.
